# A Disaggregation Strategy for Nanopesticide Fabrication: Investigating the Impact of Nanosizing on Pesticide Biointeractions

**DOI:** 10.1002/advs.75914

**Published:** 2026-06-02

**Authors:** Jiaqi Wei, Jianxin Feng, Huiting Zhu, Kunzhong Lin, Chenhan Liu, Lin Li, Zhuoyan Xiao, Daihao Huang, Rong Liu, Huipeng Pan, Lingda Zeng, Hanhong Xu

**Affiliations:** ^1^ State Key Laboratory of Green Pesticide Key Laboratory of Natural Pesticide and Chemical Biology Ministry of Education College of Plant Protection South China Agricultural University Guangzhou China

**Keywords:** bioactivity, disaggregation, emamectin benzoate, nanopesticide, plant utilization, size effect, toxicity

## Abstract

Clarifying the intrinsic impact of nanosizing on pesticide biointeractions remains challenging because most nanopesticides are formulated with nanocarriers or surfactants that may influence these interactions. Here, we report a disaggregation strategy for fabricating nanopesticides without nanocarriers or surfactants. Adding acetic acid to an emamectin benzoate (EB) suspension yielded a stable EB nanopesticide (HOAc‐EB) with an average particle diameter of 7 nm. In HOAc‐EB, acetic acid disrupted hydrogen bonding in EB rather than forming complexes with it. Nanosizing significantly increased bioactivity against *Megalurothrips usitatus* and *Meloidogyne enterolobii*, improved foliar and root penetration, and reduced soil adsorption. Compared with EB, HOAc‐EB reduced the 24‐h LC_50_ by 91% against *Megalurothrips usitatus* (3.02 to 0.280 mg·L^−1^) and by 56% against *Meloidogyne enterolobii* (18.5 to 8.1 mg·L^−1^), along with 5.1‐fold higher peak foliar penetration in cowpea and 4.4‐fold higher peak root penetration in chili pepper at 1000 mg·L^−1^. Field trials confirmed improved control of *Meloidogyne enterolobii*. HOAc‐EB was safe for crops at 1000 mg·L^−1^ and showed no greater toxicity than EB in zebrafish, earthworms, or mice. This study provides insights into the impact of nanosizing on pesticide biointeractions. The simple, eco‐friendly, and cost‐effective construction approach advances the practical production and agricultural applications of HOAc‐EB.

## Graphical abstract

To explore the impact of nanosizing on pesticide biointeractions, a 7‐nm (average) emamectin benzoate nanopesticide without nanocarriers or surfactants is fabricated via HOAc‐mediated disaggregation. Nanosizing enhances bioactivity against *Megalurothrips usitatus* and *Meloidogyne enterolobii* and improves plant penetration. Field trials confirm superior root‐knot nematode control. No increased toxicity to nontarget organisms is observed.

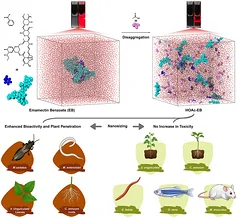

## 1 Introduction

Pesticides play a critical role in global food security by reducing crop losses caused by pests, pathogens, or weeds [1, 2, 3]. However, concerns regarding their environmental and health impacts have driven the development of advanced techniques to improve efficiency, safety, and sustainability [4, 5, 6, 7, 8, 9, 10]. Among these innovations, nanotechnology has garnered significant attention because it can endow pesticides, termed nanopesticides, with advantages such as size effects and tailorable functionalities. These systems tend to exhibit properties superior to those of conventional pesticide formulations [11, 12, 13, 14, 15, 16]. For instance, nanopesticides have been reported to achieve 59%–97% improvement in control efficacy, enhancement in spreading ability (reducing contact angles on leaves by above 40°), and even confer systemic properties to non‐systemic pesticides in various studies [17, 18, 19, 20, 21, 22, 23, 24, 25, 26]. Despite these advances, a fundamental question in this field remains unclear: the impact of nanosizing on pesticide biointeractions, such as bioactivity, plant utilization, and toxicity. Clarifying these aspects will improve understanding of the size effects of nanopesticides, which is essential for their in‐depth study and development, safety, and environmental sustainability assessment, formulation of relevant regulations, and practical adoption.

Typically, nanopesticides are constructed through loading by nanocarriers or self‐assembly with surfactants [27, 28, 29, 30, 31]. These nanocarriers or surfactants not only reduce pesticides to the nanoscale but may also impart specialized functionalities, such as targeted delivery and biosafety improvement [32, 33, 34, 35]. For instance, significant efforts have been devoted to constructing nanopesticides of emamectin benzoate (EB), a highly efficient and broad‐spectrum insecticide that has long ranked among the top ten globally, by introducing nanocarriers or surfactants. For nanocarrier‐based strategies, Li et al. employed ionic liquids as functional media to construct unimolecular nanopesticides (∼3 nm), enabling efficient dispersion and delivery of EB molecules and significantly enhancing penetration across insect cuticles and plant leaves [36]; Yang et al. constructed an EB nanopesticide (∼92 nm) with copolymer surfactant, nonionic surfactant and sodium alginate cross‐linked by Ca^2+^, achieving pH‐responsive release of EB and reducing its toxicity [37]; Huang et al. developed a dual‐coated EB nano‐formulation (∼96 nm) based on lignin and urea‐formaldehyde, which lowered the photodegradation rate and prolonged the insecticidal duration [38]. For surfactant‐based strategies, An et al. synthesized a specific surfactant to induce EB co‐assembly with it into a water‐based nanodelivery system (∼48 nm), improving stability, prolonging efficacy, and reducing toxicity [39]. These approaches enhance the efficacy, penetration, safety, and persistence of EB. However, because nanocarriers or surfactants tightly bind to EB, they may influence pesticide biointeractions through their own interactions with organisms, whereas nanosizing itself can also alter pesticide biointeractions. These overlapping effects complicate the evaluation of the intrinsic impact of nanosizing in systems containing nanocarriers or surfactants.

Herein, a disaggregation strategy is reported for fabricating nanopesticides without a nanocarrier or surfactant to elucidate the inherent changes in pesticide biointeractions induced by nanosizing. Inspired by advances in supramolecular engineering and disassembly strategies [40, 41, 42, 43, 44, 45, 46, 47, 48, 49, 50, 51, 52, 53], acetic acid (HOAc) was used to promote the dispersion of EB molecules, enabling the formation of an EB nanopesticide (HOAc‐EB). The nanosizing of HOAc‐EB originated from HOAc‐mediated perturbation of the hydrogen‐bonding interactions in EB rather than the formation of a stable complex between HOAc and EB. To determine the effects of nanosizing, several pesticide biointeractions, including bioactivity, plant utilization, and toxicity, were compared between HOAc‐EB and EB. This study is expected to provide insight into the impact of nanosizing on pesticide biointeractions and to offer a new strategy for the rational design of nanopesticides.

## 2 Results

### 2.1 Nanopesticide Formation and Nanosizing Mechanism

**FIGURE 1:**
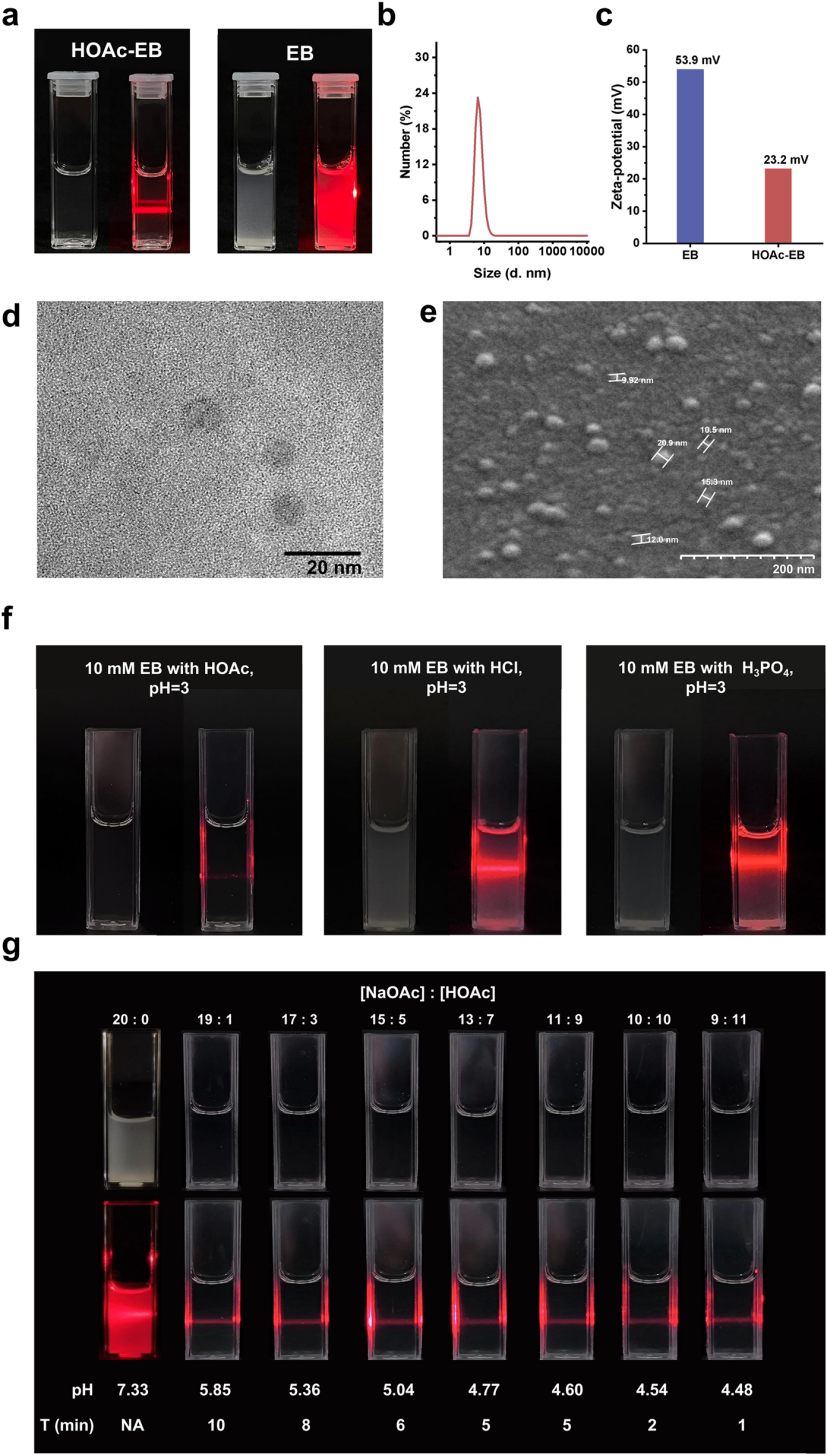
Formation of HOAc‐EB nanopesticide. (a) Photographs of HOAc‐EB (10 mm EB with 200 mm HOAc, pH = 3) and EB (10 mm). (b) DLS data of HOAc‐EB. (c) Zeta‐potential of HOAc‐EB and EB. (d) TEM and (e) cryo‐SEM image of HOAc‐EB. The total thickness of the gold layer on both sides was approximately 8 nm (on two sides), depending on the time of gold spraying. (f) Photographs of EB with HOAc, hydrochloric acid, and phosphoric acid at pH = 3. (g) Photographs, pH, and time required for dispersion of EB (10 mm) with varying NaOAc/HOAc ratios (total concentration of 200 mm).

The disaggregation of EB upon addition of HOAc was first evidenced by the visual appearance of the mixture. As shown in Figure 1a, HOAc‐EB, prepared by simply mixing EB with a 20‐fold molar excess of HOAc in water, was clear and transparent and exhibited the Tyndall effect, suggesting the formation of colloidal aggregates. In contrast, EB alone appeared turbid in water. Nanosizing of EB was further confirmed by dynamic light scattering (DLS), transmission electron microscopy (TEM), and cryogenic scanning electron microscopy (cryo‐SEM). As shown in Figure 1b,c, the average diameter of HOAc‐EB was approximately 7 nm, with a decrease in zeta potential from 53.9 mV for EB to 23.2 mV for HOAc‐EB, indicating disaggregation of EB. TEM revealed quasi‐spherical particles with a diameter of approximately 7.4 nm. (Figure 1d), and cryo‐SEM (Figure 1e) showed particles with diameters of 10–20 nm and a gold coating of approximately 8 nm. The particle sizes were consistent with those determined by DLS. These results demonstrate that EB nanopesticides were formed by simply adding HOAc.

The role of acidity in EB disaggregation was further examined by evaluating its dispersion in inorganic acids at a constant pH. As shown in Figure 1f, the addition of strong acids, such as hydrochloric acid, and moderately strong acids, such as phosphoric acid, failed to reduce the particle size of EB aggregates to the nanoscale at pH 3.0. Although EB aggregation weakened as the pH decreased (Figure S1), neither hydrochloric acid nor phosphoric acid was as effective as HOAc in disaggregating EB. These results indicate that acidity contributes to EB disaggregation but is not the sole determining factor. The effects of different ratios of HOAc and sodium acetate (NaOAc) mixtures on EB disaggregation were further investigated. As shown in Figure 1g, mixtures of HOAc and NaOAc at various ratios disrupted EB aggregation; however, the time required for disaggregation increased with decreasing HOAc concentration and increasing pH. Moreover, NaOAc alone failed to disaggregate EB. Other short‐chain alkyl acids exhibited efficacy comparable to that of HOAc in disaggregating EB (Figure S2), highlighting the significant role of the carboxylic acid group in EB nanosizing. Notably, ionic strength was not controlled in these experiments because high ionic strength did not promote EB disaggregation (Figure S3).

**FIGURE 2:**
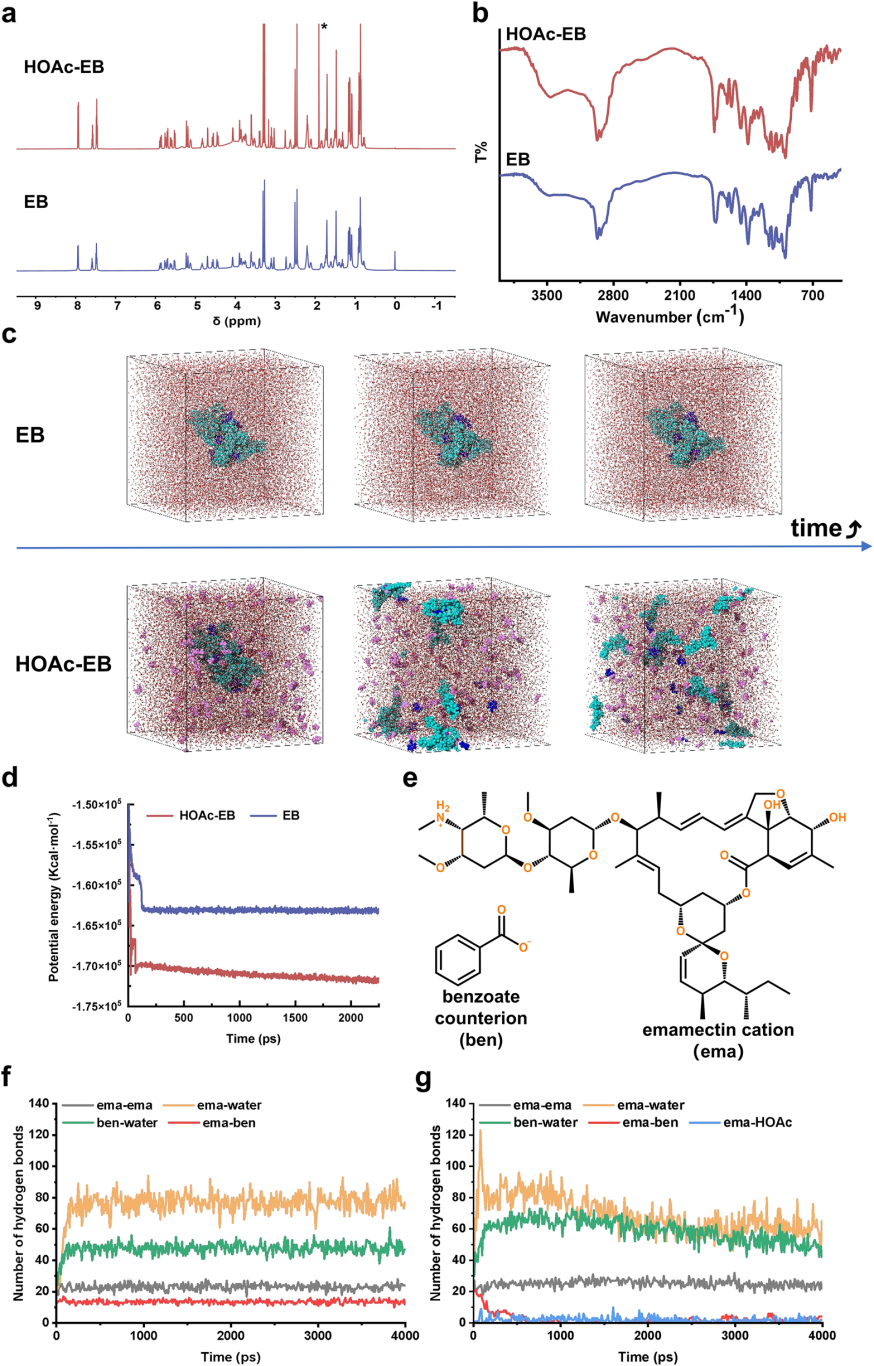
Effect of HOAc on the nanosizing of EB. (a) ^1^H NMR spectra of freeze‐dried EB and HOAc‐EB in d_6_‐DMSO. The signal marked with ^*^ corresponds to the methyl protons of HOAc in the ^1^H NMR spectrum. (b) FT‐IR spectra of freeze‐dried EB and HOAc‐EB. (c) Representative MD snapshots of EB dispersion in the EB system and HOAc‐EB system. The light blue space‐filling model refers to emamectin cation. The dark blue space‐filling model refers to the benzoate counterion. The purple space‐filling model refers to the HOAc molecule. (d) Variation in the potential energy of the EB system and the HOAc‐EB system from MD simulations. (e) Schematic molecular structure of EB, highlighting potential hydrogen bonding sites. Variation in hydrogen bond numbers between components in the (f) EB system and (g) HOAc‐EB system from MD simulations. Ema refers to emamectin cation, Ben refers to benzoate counterion.

The mechanism by which HOAc disrupts EB aggregates was first investigated using ^1^H NMR. As shown in Figure 2a, compared with EB alone, the ^1^H NMR spectrum of freeze‐dried HOAc‐EB exhibited only one additional peak corresponding to the protons of HOAc. The peak shape and chemical shifts of EB remained unchanged, suggesting that no chemical reaction occurred. This observation was further supported by the nearly identical peak profiles below 2000 cm^−^^1^ in the IR spectra (Figure 2b). In contrast, changes in the peak at approximately 3500 cm^−^^1^ suggest alterations in hydrogen bonding from EB to HOAc‐EB.

Molecular dynamics (MD) simulations were performed to elucidate the nanosizing mechanism. As shown in Figure 2c and Movies S1 and S2, EB molecules in pure water formed compact aggregates, whereas the introduction of HOAc led to gradual disaggregation. In terms of energy evolution, the EB system reached a steady potential energy state after 150 ps, indicating no further significant disaggregation (Figure 2d). In contrast, the HOAc‐EB system exhibited lower potential energy, suggesting greater thermodynamic stability, and the potential energy continued to decrease beyond 2000 ps, indicating a persistent tendency toward disaggregation. Analysis of hydrogen bonding further clarified the disaggregation mechanism. As shown in Figure 2e–g, the addition of HOAc induced significant fluctuations in the number of hydrogen bonds between emamectin cations and water molecules, reduced the number of hydrogen bonds between emamectin cations and benzoate counterions, and introduced transient hydrogen bonds between emamectin cations and HOAc molecules. These results indicate that EB aggregation is primarily mediated by benzoate counterions and that the introduction of HOAc disrupts these interactions. Moreover, HOAc perturbs hydrogen bonding between emamectin cations and water while forming unstable and fluctuating hydrogen bonds with the cations. Collectively, these effects disrupt EB aggregation and lead to nanosizing. Importantly, HOAc does not form stable assemblies with EB during this process.

### 2.2 Stability and Compatibility for Formulation Mixing

As suggested by the MD simulations, the HOAc‐EB system exhibits greater thermodynamic stability than the EB system. The evolution of HOAc‐EB particle size at different temperatures further supports this conclusion. After 14 days of storage at 0°C, 25°C, and 55°C, as well as after three freeze–thaw cycles, no EB precipitation was observed, and the particle size of HOAc‐EB remained stable (Figure S4). In addition, the incorporation of HOAc did not compromise the chemical stability of EB. After exposure to the same storage conditions, the active ingredient (A.I.; EB molecules) contents in HOAc‐EB and EB showed no significant difference, as determined by an established HPLC method for EB (Figures S5 and S6).

Moreover, the disaggregation mechanism ensures that HOAc‐EB maintains robust stability under concentration variations. The A.I. content in HOAc‐EB can reach 30% (Figure S7), which is significantly higher than the 5% typically found in conventional commercial water‐based EB formulations, such as microemulsions, according to the registration data on the China Pesticide Information Network. Upon dilution, the reduced probability of collision between EB particles ensures the dilutability of HOAc‐EB (Movie S3). Additionally, the decreased HOAc concentration and reduced interaction frequency with EB molecules after dilution exert a negligible effect on EB properties, which is critical for evaluating the impact of nanosizing on pesticide biointeractions. Furthermore, HOAc‐EB exhibits excellent compatibility with other pesticide formulations; no disruption of dispersion stability or EB precipitation was observed after mixing (Figure S8).

### 2.3 Bioactivity and Plant Utilization

**FIGURE 3:**
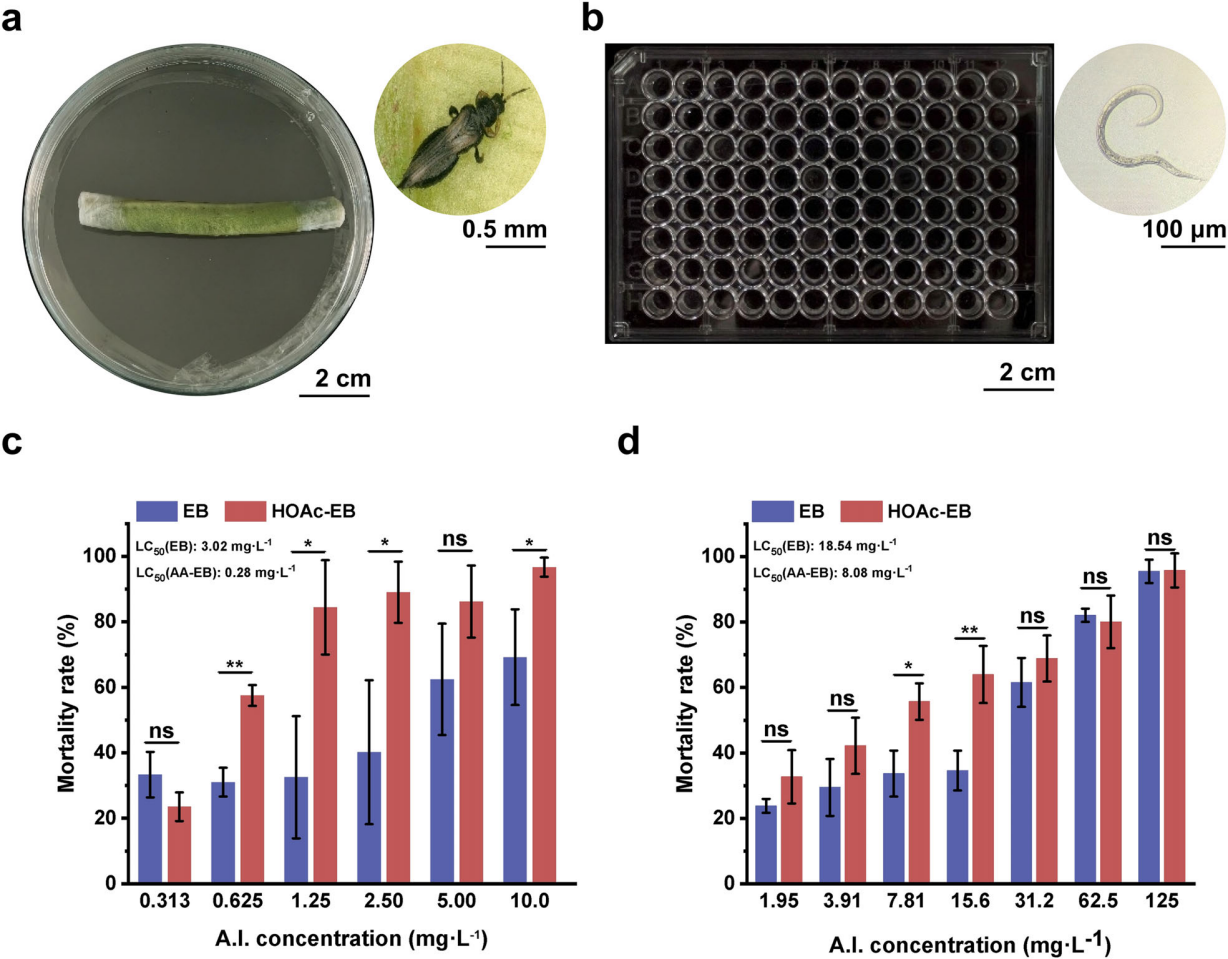
Bioactivity of HOAc‐EB nanopesticide. Bioassay setup for evaluating the activity of HOAc‐EB and EB against (a) *Megalurothrips usitatus* and (b) *Meloidogyne enterolobii*. Dose‐mortality relationship of HOAc‐EB and EB against (c) *Megalurothrips usitatus* and (d) *Meloidogyne enterolobii*. Differences were analyzed using the two‐tailed unpaired *t*‐test. Data represent mean ± SD. *n* = 3 independent experiments. ns: *p* > 0.05, ^*^: *p* < 0.05, ^**^: *p* < 0.01, ^***^: *p* < 0.001.

HOAc disrupts the aggregation of EB without forming stable assemblies with EB molecules. In addition, the bioactivity of the HOAc solution was negligible, as indicated by the bioassay results (Figure S9). This nanocarrier‐ and surfactant‐free nanopesticide enables investigation of the impact of nanosizing on pesticide biointeractions. The bioactivity of HOAc‐EB was evaluated against two representative target organisms, *Megalurothrips usitatus* and *Meloidogyne enterolobii*, which cause severe damage to cowpea and chili pepper, respectively (Figure 3a,b). As shown in Figure 3c, the LC_50_ of HOAc‐EB against *Megalurothrips usitatus* was 0.280 mg·L^−1^, representing a 91% reduction compared with EB (LC_50_ = 3.02 mg·L^−1^). Similarly, the LC_50_ of HOAc‐EB against *Meloidogyne enterolobii* was 8.1 mg·L^−1^, significantly lower than that of EB (18.5 mg·L^−1^) (Figure 3d). These results demonstrate that nanosizing enhances the bioactivity of EB against target organisms compared with the bulk form.

**FIGURE 4:**
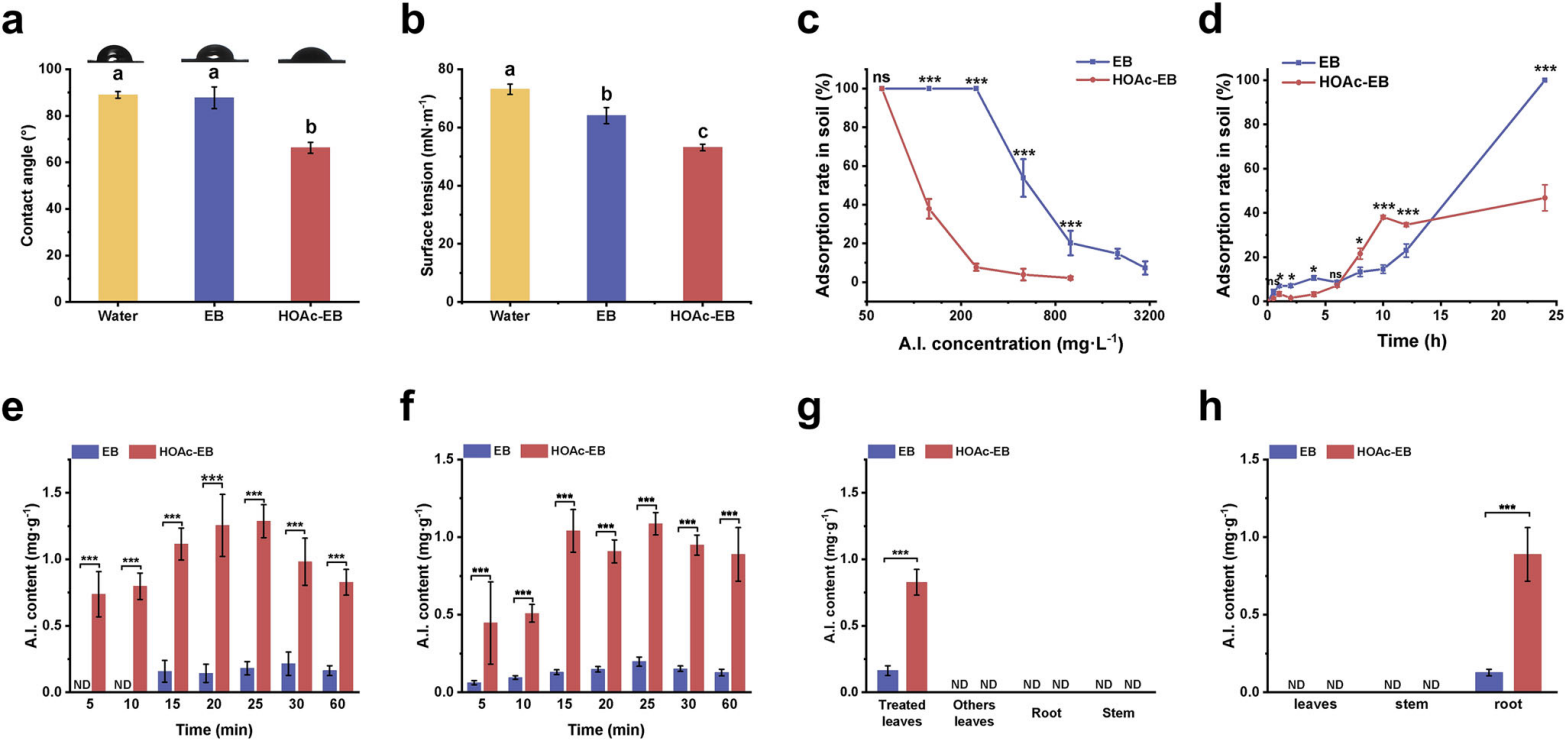
Plant utilization of HOAc‐EB nanopesticide. (a) Contact angle of HOAc‐EB, EB, and water on cowpea leaves. (b) Surface tension of HOAc‐EB, EB, and water. (c) Adsorption rate of HOAc‐EB and EB in soil with different A.I. concentration. (d) Temporal variation in adsorption rate of HOAc‐EB and EB in soil. The A.I. concentration was 150 mg/L. (e) Penetration of A.I. into cowpea leaves over time. (f) Penetration of A.I. into chili pepper roots over time. (g) A.I. content in different cowpea tissues 60 min post‐foliar application. (h) A.I. content in different chili pepper tissues 60 min post‐root immersion. Differences were analyzed using the two‐tailed unpaired *t*‐test. Data represent mean ± SD. *n* = 3 independent experiments. ns: *p* > 0.05, ^*^: *p* < 0.05, ^**^: *p* < 0.01, ^***^: *p* < 0.001.

In addition to bioactivity, plant utilization of pesticides is critical for effective pest and disease control. To evaluate the effect of nanosizing on plant utilization of EB, the spreading behavior of pesticide droplets on leaf surfaces was first investigated. As shown in Figure 4a, the contact angle of HOAc‐EB on cowpea leaves was 66.3°, significantly lower than that of EB (87.8°). This difference may be attributed to the increased surface activity of the HOAc‐EB system, as reflected by its lower surface tension (53.1 mN·m^−1^) compared with that of the EB system (64.1 mN·m^−1^; Figure 4b). Although this effect may be less pronounced than that of conventional surfactants, nanosizing still improves droplet spreading of EB on plant surfaces.

For root‐drenching applications, the ability of pesticides to avoid strong soil adsorption is a prerequisite for plant root uptake. Therefore, the adsorption behavior of HOAc‐EB in soil was investigated. As shown in Figure 4c, EB was completely adsorbed by soil within 24 h at concentrations below 250 mg·L^−1^ A.I. In contrast, HOAc‐EB was fully adsorbed only at 62.5 mg·L^−1^ A.I., and its adsorption rate decreased to below 10% at 250 mg·L^−1^ A.I. Furthermore, compared with EB, HOAc‐EB exhibited enhanced desorption from soil (Figure S10). These observations may be attributed to the increased water solubility of nanoparticles after nanosizing, as explained by the Kelvin effect. Soil adsorption kinetics experiments further support this interpretation. As shown in Figure 4d, HOAc‐EB entered the rapid adsorption phase earlier than EB.

The penetration and transport behaviors of pesticides determine their utilization in different plant tissues. EB exhibits translaminar but not systemic properties; it can penetrate the plant surface to reach internal tissues but does not translocate within the plant. After nanosizing, the translaminar movement of EB in plants was significantly enhanced. As shown in Figure 4e, localized application on cowpea leaves revealed that the peak accumulated A.I. content for HOAc‐EB (1.29 mg·g^−1^ at 25 min) was 5.1‐fold higher than that for EB (0.21 mg·g^−1^ at 30 min). Rootward penetration experiments further confirmed this enhancement. As shown in Figure 4f, when chili pepper roots were immersed in HOAc‐EB, the peak accumulated A.I. content (1.09 mg·g^−1^ at 25 min) was 4.4‐fold higher than that for EB (0.20 mg·g^−1^ at 25 min). Regarding systemic behavior, whether from root to leaf or leaf to root, A.I. was detected only at the application site for both HOAc‐EB and EB (Figure 4g,h). These results suggest that penetration and transport behaviors are primarily governed by molecular properties, whereas particle size mainly influences the extent of these processes. Nanosizing enhances the bioactivity and translaminar properties of EB but does not confer systemic behavior that it inherently lacks.

**FIGURE 5:**
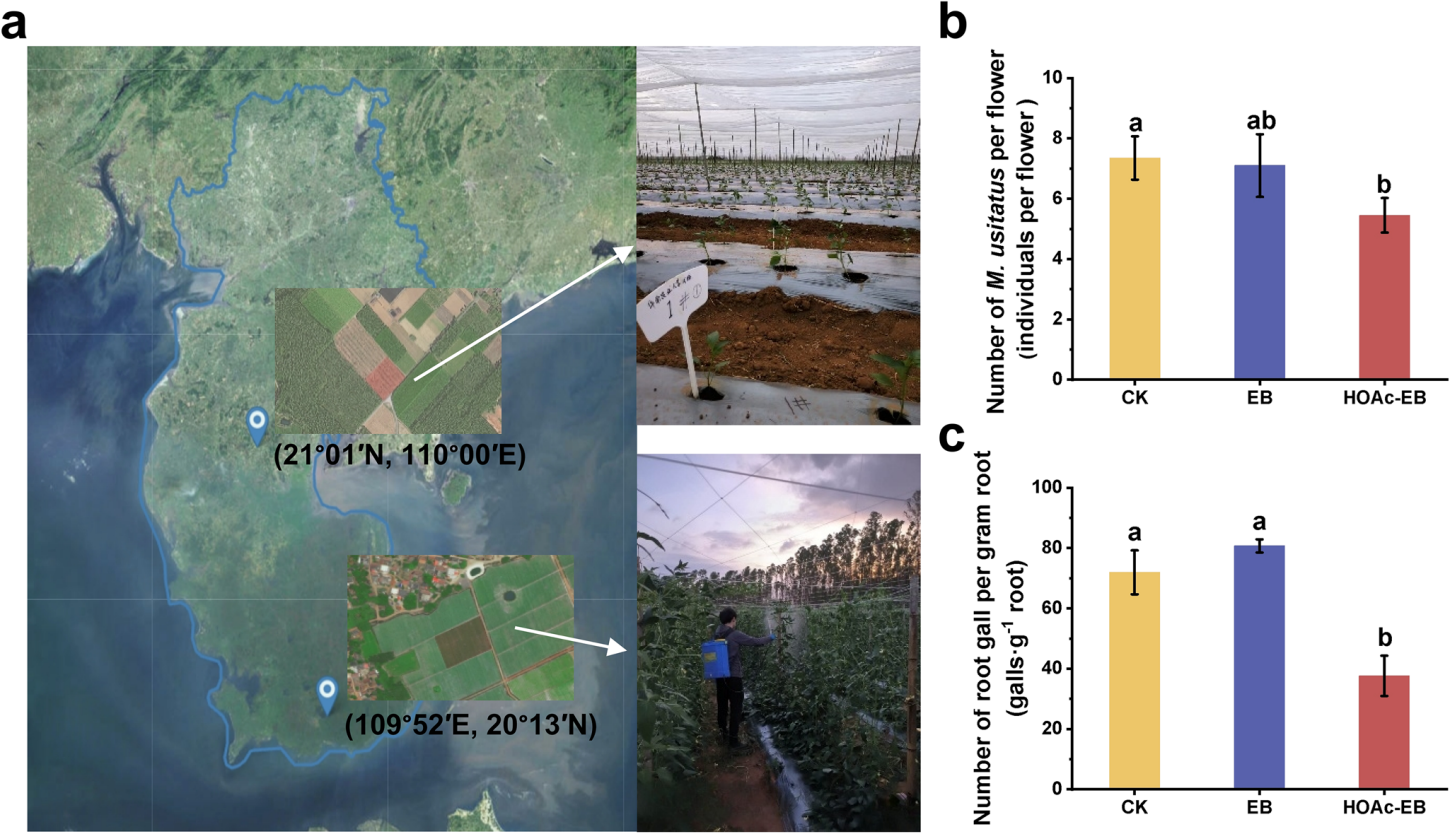
Field Performance of HOAc‐EB nanopesticide. (a) Illustration of field trial site and trial scenarios. (b) Field control efficacy against *Megalurothrips usitatus* on cowpea. *n* = 4 independent experiments. (c) Field control efficacy against *Meloidogyne enterolobii* on chili pepper. *n* = 3 independent experiments. Differences were analyzed using one‐way ANOVA and Tukey's post hoc test, with different lowercase letters indicating significant differences (*p* < 0.05). Data represent mean ± SD.

Field trials were conducted to determine whether the increased bioactivity and plant utilization of HOAc‐EB translate into improved efficacy for pest and disease control compared with EB (Figure 5a). As shown in Figure 5b, in the control of *Megalurothrips usitatus* on cowpea, the number of thrips per flower decreased to 5.5 individuals per flower after 48 h of HOAc‐EB treatment, compared with 7.1 individuals per flower in the EB group. However, statistical analysis indicated no significant difference between the HOAc‐EB and EB groups. In contrast, efficacy against *Meloidogyne enterolobii* was markedly improved. The root gall count in the EB‐treated group (80.7 galls·g^−1^ root) was statistically indistinguishable from that in the untreated control (72.0 galls·g^−1^ root), likely due to strong soil adsorption. In comparison, HOAc‐EB reduced the gall count to 37.6 galls·g^−1^ root, transforming EB from ineffective to effective in controlling *Meloidogyne enterolobii* (Figure 5c). Although nanosizing significantly improves bioactivity and plant utilization under laboratory conditions, these advantages may not consistently translate into enhanced field efficacy. Nanoscale size alone is not sufficient to address field pest and disease challenges; additional functionalities, such as reduced soil adsorption, must also be considered.

### 2.4 Toxicity

**FIGURE 6:**
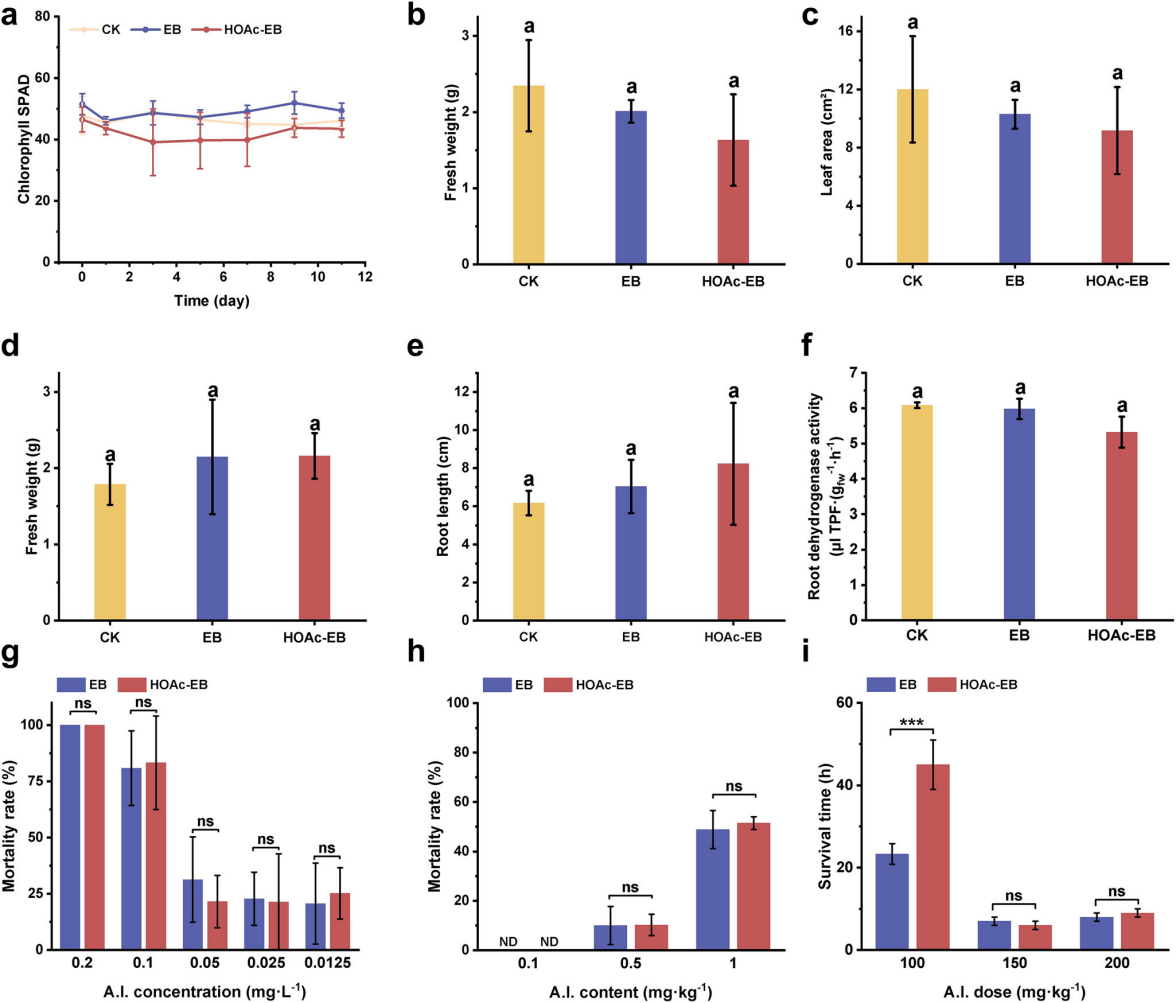
Biosafety assessment of HOAc‐EB nanopesticide on nontarget organisms. (a) Temporal variation in chlorophyll content in leaves of cowpea plants sprayed with HOAc‐EB, EB, or water. (b) Fresh weight and (c) leaf area of cowpea plants sprayed with HOAc‐EB, EB, or water for 14 days. Treatments for panels (a–c) were applied consecutively at 9:00 AM daily. (d) Fresh weight, (e) root length, and (f) root dehydrogenase activity of chili pepper plants root‐drenched with HOAc‐EB, EB, or water after 14 days. Treatments for panels (d–f) were applied on the first day. (g) Dose‐mortality relationship of HOAc‐EB and EB against zebrafish at 24 h. (h) Dose‐mortality relationship of HOAc‐EB and EB against Earthworm at 5 d. (i) Survival time of mice treated with different doses of HOAc‐EB or EB. Differences in (b–f) were analysed using one‐way ANOVA and Tukey's post hoc test, with different lowercase letters indicating significant differences (*p* < 0.05). Differences in (g–i) were analyzed using the two‐tailed unpaired *t*‐test. Data represent mean ± SD. *n* = 3 independent experiments. ns: *p* > 0.05, ^*^: *p* < 0.05, ^**^: *p* < 0.01, ^***^: *p* < 0.001.

Nanosizing increases the efficacy of EB against target organisms; however, its impact on toxicity toward nontarget organisms remains a critical concern. To address this, the toxicity of HOAc‐EB and EB to plants and animals was compared, with dosing selected according to established references [36, 39, 54, 55]. In a phytotoxicity assessment based on foliar spraying of cowpea leaves, repeated applications of HOAc‐EB at a high concentration of 1000 mg·L^−1^ over 14 days did not induce visible phytotoxic symptoms (Figure S11). No significant differences in plant physiological parameters, including total leaf chlorophyll content (Figure 6a), fresh weight (Figure 6b), and leaf area (Figure 6c), were observed between the treatment and control groups. Phytotoxicity following root drenching with HOAc‐EB was also evaluated in chili pepper plants. As shown in Figure S12, a single high‐concentration root drench (20 mL, 1000 mg·L^−1^) caused no visible growth inhibition or morphological changes over a 14‐day observation period. In terms of fresh weight and root length, neither EB nor HOAc‐EB inhibited plant growth (Figure 6d,e). Root viability further indicated comparable dehydrogenase activity between the HOAc‐EB treatment and control groups (Figure 6f), confirming the absence of root phytotoxicity. Despite the nanosizing‐induced increase in EB penetration into cowpea leaves and chili pepper roots, no phytotoxic effects were observed. Measurements of malondialdehyde content and the activities of superoxide dismutase, catalase, and peroxidase in cowpea and chili pepper indicated that both EB and HOAc‐EB treatments induced mild stress at 1000 mg·L^−1^ A.I. (Figure S13).

To assess toxicity toward nontarget animals, zebrafish, earthworms, and mice were selected as test organisms. Acute toxicity assays in zebrafish revealed comparable 24 and 48h toxicity between HOAc‐EB and EB (Figure 6g; Figure S14). In earthworm contact toxicity tests, no significant differences were observed between HOAc‐EB and EB after 14 days of exposure (Figure 6h). Oral acute toxicity experiments in mice showed no differences between HOAc‐EB and EB at doses of 150 and 200 mg·kg^−1^. However, at 100 mg·kg^−1^, the survival time of mice in the HOAc‐EB group increased to 45.0 h, significantly longer than the 23.3 h observed in the EB group (Figure 6i). These findings indicate that nanosizing does not exacerbate the toxicity of EB. The selective enhancement of HOAc‐EB activity toward target organisms, rather than nontarget organisms, may arise from differences in uptake and metabolism or from altered affinity for target protein binding sites, rather than from increased nonspecific toxicity.

## 3 Discussion and Conclusion

HOAc enables the formation of a stable colloidal dispersion with an average particle size of 7 nm by disrupting the intermolecular hydrogen‐bonding network of EB molecules. During this nanosizing process, HOAc does not form complexes with EB but effectively minimizes interference from formulation additives, thereby enabling systematic investigation of the impact of nanosizing on pesticide biointeractions. Nanosizing enhances the bioactivity of EB against target organisms without increasing toxicity to nontarget organisms, with LC_50_ values reduced by 91% for *Megalurothrips usitatus* and 56% for *Meloidogyne enterolobii*. In terms of plant utilization, nanosizing improves the translaminar movement of EB while preserving its non‐systemic nature, with peak penetration increasing by 5.1‐fold in cowpea leaves and 4.4‐fold in chili pepper roots. In addition, nanosizing reduces soil adsorption of EB and promotes improved droplet distribution on plant surfaces. However, these advantages do not fully translate into enhanced field efficacy. In field trials, HOAc‐EB demonstrated significantly greater control efficacy against *Meloidogyne enterolobii* than EB but did not significantly improve control of *Megalurothrips usitatus*.

These results indicate that although small size is important for nanopesticides, other factors—such as charge and its distribution, hydrophobicity/hydrophilicity, dissolution behavior, and overall transformation potential—also play critical roles in determining nanopesticide fate and efficacy. Dynamic regulation of surface charge can simultaneously improve foliar deposition and cellular uptake [56]. In addition, microenvironment‐triggered protonation, decomposition, or enzyme‐responsive disassembly can alter dissolution, release, and transformation processes, thereby reshaping bioavailability and target interaction [57]. Surface affinity‐driven capture mechanisms further show that nanopesticide efficacy depends not only on nanoscale dimensions, but also on charge‐related interfacial behavior, hydrophobicity/hydrophilicity‐associated surface performance, and overall transformation potential [58]. Compared with recently reported systems, this disaggregation approach enables nanosizing of EB without nanocarriers or surfactants. It thus isolates the size effect from formulation‐related interferences and allows a more direct evaluation of how nanosizing influences bioactivity, plant utilization, and toxicity. The results demonstrate that nanosizing alone achieves enhanced bioactivity and tissue penetration but cannot universally address field pest and disease challenges, highlighting the importance of integrating size effects with additional functionalities in future designs.

In terms of economic feasibility and production scalability, the HOAc‐EB system offers advantages that mitigate concerns regarding cost and manufacturing complexity. Under the formulation conditions used in this study (molar ratio of EB to HOAc = 1:20), preparation of a formulation containing 1.0 kg of A.I. requires approximately 1.2 kg of HOAc. Based on recent bulk market prices for glacial HOAc in China, this corresponds to an additional auxiliary material cost of only 2.6–3.4 Chinese yuan per kilogram of A.I. Given that the market price of technical EB in China is approximately 630–640 Chinese yuan per kilogram, the incremental cost of HOAc represents only ∼0.5% of the A.I. cost. Beyond the minimal material cost, this strategy eliminates the need for energy‐intensive high‐shear grinding, organic solvents, and exogenous surfactants, thereby reducing associated capital and operational expenditures. In addition, the formulation achieves an A.I. loading of up to 30%, which lowers packaging, storage, and transportation costs. From a manufacturing perspective, the HOAc‐EB system is formed by simple mixing, undergoing spontaneous dispersion without the need for specialized or high‐energy equipment. Scale‐up is highly feasible, as the process involves a straightforward mass‐transfer operation requiring only corrosion‐resistant stirred vessels, without the heat‐transfer limitations or pressure constraints typical of more complex synthetic routes. Collectively, these factors indicate that the enhanced efficacy achieved through HOAc‐mediated disaggregation of EB into a nanopesticide architecture is not offset by prohibitive production costs or scale‐up barriers, thereby supporting the commercial relevance and industrial applicability of this strategy.

In conclusion, a disaggregation strategy was developed to fabricate a nanopesticide (HOAc‐EB) without nanocarriers or surfactants. This system provides insights into the size effects of nanopesticides, which are essential for advancing their in‐depth study and development. Compared with EB, HOAc‐EB exhibits notable advantages, including high stability and compatibility for formulation mixing, enhanced bioactivity and plant utilization efficiency, and no increase in toxicity. Some of these advantages translate into improved field efficacy, as demonstrated by the higher efficacy of HOAc‐EB in controlling *Meloidogyne enterolobii*. Importantly, HOAc‐EB production involves a streamlined process—simply mixing EB with HOAc, a biocompatible, cost‐effective, and readily available compound—without the need for energy‐intensive grinding or the use of surfactants or organic solvents to achieve dispersion and stabilization. This approach offers overall benefits, including reduced processing complexity, minimized environmental impact, and improved scalability, thereby highlighting its potential for practical production and agricultural applications.

## 4 Materials and Methods

### 4.1 Chemicals and Materials

All chemicals were obtained from commercial suppliers and used without further purification. Acetic acid (HOAc, AR, catalogue no. A801295‐500 mL) was purchased from Shanghai Macklin Biochemical Technology Co., Ltd. Emamectin benzoate (EB, B1a, 97%) was obtained from Jingman Jinxianda Biotechnology Co., Ltd. HOAc‐EB was prepared by simply mixing EB with HOAc in water. Chlorantraniliprole suspension concentrate (30%) was purchased from Shandong Guihe Biotechnology Co., Ltd. Indoxacarb suspension concentrate (150 g·L^−1^) was obtained from FMC Corporation (USA). Tebufenozide suspension concentrate (20%) was acquired from Shandong Caoda Chemical Co., Ltd. Tetrazolium red (98%, catalogue no. PH1792) was purchased from Fuzhou Phygene Biotechnology Co., Ltd.

*Megalurothrips usitatus* was collected from Fenyong Farm, Leizhou City, Guangdong Province (21°01′ N, 110°00′ E) and maintained in an incubator at 26°C ± 1°C with 75% ± 5% relative humidity and a 16 h photoperiod for 72 h.

*Meloidogyne enterolobii* was collected from infected chili roots in Qianshan Town, Zhanjiang City, Guangdong Province (109°52′ E, 20°13′ N). Egg masses were manually dissected from infected chili roots using forceps and transferred to a Petri dish containing a sieve covered with a paper towel and sterile water. The dish was incubated at 25°C under dark conditions. Second‐stage juveniles were collected 2–3 days later using a modified Baermann funnel method and stored at 4°C for subsequent experiments.

Earthworms (*Eisenia fetida*) were procured from Binzhou Hengda Sihai Trading Co., Ltd. AB strain zebrafish (*Danio rerio*) were procured from Hangzhou Hante Biotech Co., Ltd. Prior to toxicity assays, zebrafish were acclimated for 3 days in dechlorinated, aerated water at 28°C (14 h light/10 h dark photoperiod) and fed once daily with a commercial diet. Only healthy zebrafish exhibiting normal swimming behavior, intact morphology, and no visible lesions were selected. Before exposure, earthworms were maintained in moistened soil or substrate at 20°C for 24 h. Active individuals with normal appearance, intact body walls, and no visible injury were used. BALB/c mice (*Mus musculus*) were obtained from Shenzhen Rongwan Biomedical Laboratory Animal Center. Mice were housed under standard laboratory animal husbandry conditions at 22°C and 50% relative humidity under a 12 h light/12 h dark cycle, with free access to standard chow and water. Mice were acclimated for at least 7 days before dosing, and only healthy animals were included in the experiments.

Animal experiments were performed in accordance with the ethical guidelines of the South China Agricultural University Laboratory Animal Ethics Committee (earthworm, Application No. 2024G041; zebrafish, Application No. 2024G040) and Shenzhen Glorybay Biotech Co., Ltd. (mice, Approval No. RW‐IACUC‐24‐0124).

Cowpea (*Vigna unguiculata*) plants were sourced from Jieyang Nongyan Seed Industry Co., Ltd. Chili pepper (*Capsicum annuum*) plants were procured from Shouguang Xinxinran Horticulture Co., Ltd.

### 4.2 Instruments

Dynamic light scattering (DLS) and zeta potential measurements were performed using a Zetasizer Nano ZSE instrument (Malvern Panalytical Ltd., UK) at an EB concentration of 10 mm. Cryogenic scanning electron microscopy (cryo‐SEM) analysis was conducted using a Regulus 8100 microscope (Hitachi High‐Technologies, Japan) at an EB concentration of 100 mg·L^−1^, with gold sputter coating for 40 s. Nuclear magnetic resonance (NMR) spectra were acquired using an AVANCE III HD 600 MHz spectrometer (Bruker Corporation, Germany). Fourier‐transform infrared (FT‐IR) spectra were recorded using a Nicolet iS10 spectrometer (Thermo Fisher Scientific, USA). For HOAc‐EB samples, ^1^H NMR and FT‐IR spectra were obtained from solutions prepared at an EB concentration of 10 mm, which were then freeze‐dried prior to analysis. Contact angle and surface tension measurements were performed using an OCA20 goniometer (DataPhysics Instruments, Germany) at an EB concentration of 500 mg·L^−1^ (contact angle test liquid volumes: 4 and 10 µL). High‐performance liquid chromatography (HPLC) analysis was carried out using a Waters 2545 Binary Gradient Module with a Waters 2998 Photodiode Array Detector and a Waters Atlantis T3 column (4.6 mm × 250 mm, 5 µm). Methanol was used as mobile phase A, and 0.02% formic acid aqueous solution was used as mobile phase B (VA:VB = 4:6). The operating conditions were as follows: flow rate, 0.8 mL·min^−1^; injection volume, 10 µL; detection wavelength, 235 nm; and column temperature, 30°C.

### 4.3 Transmission Electron Microscopy (TEM) Measurement

TEM was performed using a Talos F200S microscope (FEI, USA). A 10 µL aliquot of HOAc‐EB (EB concentration: 100 mg·L^−1^) was dropped onto a 300‐mesh ultrathin carbon‐coated copper grid. After standing at room temperature for 30 min, the excess solution was removed. The sample was negatively stained with 2% phosphotungstic acid hydrate for 5 min and then dried prior to TEM observation and imaging.

### 4.4 Molecular Dynamics (MD) Simulations

Two EB clusters, each comprising 10 EB molecules, were constructed in two distinct systems: one in pure water (EB system) and the other in a mixed HOAc–water environment (HOAc‐EB system). The clusters were centered in an MD simulation box with dimensions of 95 × 95 × 95 Å^3^. For the EB system, 20 000 water molecules were placed in the simulation box, whereas for the HOAc‐EB system, 20 000 water molecules and 100 HOAc molecules were included. Periodic boundary conditions (PBCs) were applied in all three orthogonal directions to eliminate surface effects. Atomic interactions in both systems were modeled using the Polymer Consistent Force Field (PCFF) [59]. Non‐bonded interactions were calculated as follows: van der Waals forces were described using the Lennard‐Jones 9‐6 potential with a 10 Å cutoff, while long‐range electrostatic interactions were computed using the Coulomb potential with the particle–particle–particle–mesh (PPPM) algorithm. Prior to MD simulations, both systems were energy‐minimized to a local minimum with energy and force tolerances of 1.0 × 10^−4^ kcal·mol^−1^ and 1.0 × 10−^4^ kcal·mol^−1^·Å^−1^, respectively. Subsequently, the minimized structures were relaxed at 300 K and 1 atm under an isobaric‐isothermal (NPT) ensemble for 100 000 timesteps. Finally, MD simulations were performed for 2 250 000 timesteps at 300 K under an NVT ensemble to evaluate system stability. Atomic motion in both systems followed classical Newtonian mechanics. The velocity–Verlet algorithm with a timestep of 1.0 fs was used to integrate Newton's equations of motion. All MD simulations were carried out using the Large‐scale Atomic/Molecular Massively Parallel Simulator (LAMMPS) package [60].

### 4.5 Thermodynamic Stability of HOAc‐EB

EB (1.008 g) was weighed and dispersed in 100 mL of deionized water to obtain a 10 mm EB suspension. HOAc (1.201 g) was added at an EB:HOAc molar ratio of 1:20, and the mixture was vortexed for 30 s to prepare the HOAc‐EB suspension. Subsequently, 5 mL aliquots were transferred into 50 mL conical vials and stored separately at 0°C (freezer), 25°C (incubator), and 55°C (oven). The properties of the HOAc‐EB suspension were evaluated using the following analyses: (1) EB content quantification by high‐performance liquid chromatography (HPLC) and visual inspection of appearance on days 0, 2, 4, 6, 8, 10, 12, and 14; and (2) particle size determination by dynamic light scattering (DLS) on day 14. For freeze–thaw stability testing, samples were frozen at −20°C for 24 h and then thawed at 25°C for 24 h; this cycle was repeated three times. After each cycle, appearance, particle size, and EB content were analyzed. A 10 mm EB suspension was used as the control group and stored under the same temperature conditions and freeze–thaw cycles as the HOAc‐EB samples. After each storage period or freeze–thaw cycle, the suspensions were vortexed, and EB content was determined by HPLC.

### 4.6 Mixing Compatibility of HOAc‐EB With Three Pesticide Formulations

According to common co‐formulation ratios, three pesticide mixtures were prepared using HOAc‐EB: (1) 9% chlorantraniliprole + 3% EB, (2) 2% indoxacarb + 1% EB, and (3) 12% tebufenozide + 3% EB. These mixtures were diluted according to recommended field application rates. Specifically, the 9% chlorantraniliprole + 3% EB mixture was diluted 3000‐fold, the 2% indoxacarb + 1% EB mixture was diluted 500‐fold, and the 12% tebufenozide + 3% EB mixture was diluted 3000‐fold, resulting in three commonly co‐formulated pesticide suspensions. The appearance of the mixtures was observed at 0 and 2 h. The particles in the suspension components were examined using an optical microscope. A 1 mL aliquot of supernatant was filtered through a 0.22 µm nylon membrane filter, and the EB content in the filtrate was determined by HPLC.

### 4.7 Indoor Bioassay of HOAc‐EB Against *Megalurothrips Usitatus*

*Megalurothrips usitatus* individuals with strong vitality and no visible external injuries were collected and selected for the experiment. Fresh cowpea pods were harvested, thoroughly washed, air‐dried, and cut into approximately 1 cm segments for subsequent use. HOAc solutions were prepared at concentrations of 50, 100, and 200 mg·L^−1^. HOAc‐EB and EB dispersions with A.I. concentrations of 0.625, 1.25, 2.5, 5, and 10 mg·L^−1^ were prepared, respectively. Prior to treatment, thrips were starved for 2 h. The pre‐cut cowpea segments were then immersed in the respective test dispersions for 15 s, removed, and allowed to air‐dry naturally. Each segment was placed into a Petri dish (35 mm diameter) lined with filter paper, and approximately 20 *Megalurothrips usitatus* individuals were introduced into each dish. The dishes were sealed with breathable medical gauze and incubated under controlled conditions at 28°C. After 24 h, mortality was recorded. During evaluation, thrips were gently touched with a fine brush; individuals were considered dead if no voluntary movement or appendage response was observed. The experiment was independently repeated three times.

### 4.8 Indoor Bioassay of HOAc‐EB Against *Meloidogyne Enterolobii*

Second‐stage juveniles (J2s) of *Meloidogyne enterolobii* were counted under a stereomicroscope. *Meloidogyne enterolobii* suspensions were prepared at a concentration of approximately 200 individuals per 100 µL. HOAc solutions were prepared at concentrations of 100, 200, and 400 mg·L^−1^, while HOAc‐EB and EB dispersions were prepared at A.I. concentrations of 1.95, 3.91, 7.81, 15.6, 31.2, 62.5, and 125 mg·L^−1^. In a 96‐well microtiter plate, each well received 100 µL of pesticide dispersion and 100 µL of *M. enterolobii* suspension (1:1 ratio), which were then thoroughly mixed. Sterile water served as the blank control. The plates were incubated under humidified conditions at 25°C. After 24 h, the mortality of *M. enterolobii* was assessed under a stereomicroscope. The experiment was independently repeated three times.

### 4.9 Soil Adsorption Properties

The soil used in this study was light clay collected from Zhanjiang, Guangdong, China, with a pH of 4.1 ± 0.1, an organic matter content of 16.0 ± 0.1, and a cation exchange capacity of 5.7 ± 0.1.

#### 4.9.1 Soil Adsorption Experiment

Soil samples (2 g) collected from Zhanjiang, Guangdong (0–5 cm depth) were weighed and placed in 100 mL Erlenmeyer flasks. Subsequently, 20 mL of HOAc‐EB suspension at five concentrations (62.5, 125, 250, 500, and 1000 mg·L^−1^ A.I.) or EB suspension at seven concentrations (62.5, 125, 250, 500, 1000, 2000, and 3000 mg·L^−1^ A.I.) were added to each flask, with 0.01 mol·L^−1^ CaCl_2_ used as the background electrolyte. A blank control without soil was included. The flasks were sealed and incubated on a constant‐temperature shaker at 25°C (180 rpm). After 24 h, aliquots were collected and centrifuged at 4436 × g for 10 min. The supernatant was filtered through a 0.45 µm aqueous membrane filter, and the EB concentration was quantified by HPLC. The experiment was independently repeated three times.

#### 4.9.2 Soil Desorption Experiment

Soil samples (2 g) collected from Zhanjiang, Guangdong (0–5 cm depth) were weighed and placed in 100 mL Erlenmeyer flasks. Subsequently, 20 mL of HOAc‐EB or EB suspension at concentrations of 200 and 400 mg·L^−1^ A.I. were added to each flask, with 0.01 mol·L^−1^ CaCl_2_ serving as the background medium. A blank control without soil was included. The flasks were sealed and incubated on a constant‐temperature shaker at 25°C (180 rpm). After 24 h, aliquots were collected and centrifuged at 4436 × g for 10 min. The supernatant was completely removed, and 20 mL of 0.01 mol·L^−1^ CaCl_2_ solution was added to each flask. The flasks were resealed and incubated at 25°C and 180 rpm. After 24 h, aliquots were collected and centrifuged at 4436 × g for 10 min. The supernatant was filtered through a 0.45 µm aqueous membrane filter, and the EB concentration was quantified by HPLC. The experiment was independently repeated three times.

#### 4.9.3 Soil Adsorption Kinetics Experiment

Soil samples (2 g) collected from Zhanjiang, Guangdong (0–5 cm depth) were weighed and placed in 100 mL Erlenmeyer flasks. Subsequently, 20 mL of HOAc‐EB or EB suspension at a concentration of 150 mg·L^−1^ active ingredient (A.I.) was added to each flask, with 0.01 mol·L^−1^ CaCl_2_ serving as the background medium. A blank control without soil was included. The flasks were sealed and incubated on a constant‐temperature shaker at 25°C (180 rpm). Aliquots were collected at 0, 0.5, 1, 2, 4, 6, 8, 12, and 24 h. After centrifugation at 4436 × g for 10 min, the supernatant was filtered through a 0.45 µm aqueous membrane filter. The EB concentration was then quantified by HPLC. The experiment was independently repeated three times.

### 4.10 Penetration and Transport Behaviors

Healthy cowpea plants of uniform size (seedling stage, approximately 25 cm in height), with no visible pest or disease symptoms, were selected. A 50 mL centrifuge tube cap was used to mark the center of each leaf with dye to ensure a consistent application area for pesticide treatment. Subsequently, 20 µL of 1000 mg·L^−1^ (A.I.) HOAc‐EB or EB suspension was evenly applied to the marked leaf area. At 0, 10, 15, 20, 25, and 30 min post‐treatment, the marked leaf sections were collected. At 60 min post‐treatment, the marked leaf sections, as well as other leaves, stems, and roots, were collected. The harvested tissues were surface‐washed, homogenized, extracted, and filtered, followed by HPLC analysis for EB quantification. The experiment was independently repeated three times.

Chili pepper seedlings (seedling stage, approximately 25 cm in height) were immersed in 50 mL of 1000 mg·L^−1^ (A.I.) HOAc‐EB or EB suspension. At 0, 10, 15, 20, 25, and 30 min post‐treatment, root samples were collected. At 60 min post‐treatment, roots, stems, and leaves were collected. The harvested tissues were surface‐washed, homogenized, extracted, and filtered, followed by HPLC analysis for EB quantification. The experiment was independently repeated three times.

### 4.11 Field Trial

#### 4.11.1 Field Trial for *Megalurothrips Usitatus* Control on Cowpea

The field experiment was conducted at Fengyong Farm in Leizhou City, Guangdong Province (110°01′ E, 21°01′ N). HOAc‐EB was directly diluted with water to an active ingredient concentration of 10 mg·L^−1^ for application (pH = 5). EB was first dissolved in dimethyl sulfoxide (DMSO) and then diluted with water to an active ingredient concentration of 10 mg·L^−1^ for application. Water was used as the control. Each treatment was applied to an independent plot consisting of a 5 m row. Spraying was carried out at 6:00 AM. At 48 h post‐treatment, five randomly selected flowers per plot were examined, and the number of *Megalurothrips usitatus* individuals was recorded. The experiment was independently repeated four times.

#### 4.11.2 Field Trial for *Meloidogyne Enterolobii* Control on Chili Pepper

The field experiment was conducted in a chili pepper plantation in Qianshan Town, Xuwen County, Zhanjiang City, Guangdong Province (110°01′ E, 21°01′ N). The control efficacy against *Meloidogyne enterolobii* was evaluated using a root‐drenching method. The application dose was 10 mg A.I. per plant (1000 mL). HOAc‐EB was directly dispersed in water. EB was first dissolved in DMSO and then diluted with water. Water was used as the control. Each treatment was applied to the root zone of chili pepper plants, with three replicates per treatment. One month after treatment, root gall numbers were counted. The experiment was independently repeated three times.

### 4.12 Phytotoxicity Assessment

Healthy cowpea plants of uniform size (seedling stage, approximately 25 cm in height), with no visible pest or disease symptoms, were selected. Cowpea leaves were sprayed with 0.3 mL of HOAc‐EB or EB suspensions at an A.I. concentration of 1000 mg·L^−1^, while the control group received an equivalent volume of deionized water. Treatments were applied for 14 consecutive days at 9:00 AM. During the treatment period, total chlorophyll content was measured using a TYS‐B chlorophyll meter at multiple points on the leaf blade (avoiding veins). On day 14, fresh weight and leaf area were measured, and leaf tissues were sampled to determine malondialdehyde (MDA) content and antioxidant enzyme activities, including superoxide dismutase (SOD), catalase (CAT), and peroxidase (POD), using commercial kits (Yirong Dacheng Biotechnology, Shaanxi, China). The experiment was independently repeated three times.

Chili pepper plants (seedling stage, approximately 25 cm in height) were treated with 20 mL of 1000 mg·L^−1^ (A.I.) HOAc‐EB or EB suspensions via root drenching, while the control group received an equivalent volume of deionized water. On day 14, oxidoreductase activity in chili pepper roots was measured using the triphenyl tetrazolium chloride (TTC) method. Root fresh weight and root length were also determined. In addition, MDA content and antioxidant enzyme activities, including SOD, CAT, and POD, in root tissues were assayed. The experiment was independently repeated three times.

#### 4.12.1 TTC Method

Root dehydrogenase activity in chili pepper was assessed using the TTC method. A 0.1% (w/v) TTC solution was prepared by dissolving 0.0335 g of TTC powder in distilled water and diluting to a final volume of 100 mL in a volumetric flask. Uniformly grown root samples were rinsed with deionized water and surface‐dried. A 1.000 g root sample (fresh weight, fw) was accurately weighed, immersed in the TTC solution, and incubated in the dark at 25°C ± 2°C for 24 h. After incubation, root samples were rinsed 3–5 times with distilled water, surface‐dried, and homogenized in 5 mL of acetone. The homogenate was centrifuged at 4436 × g for 10 min. The absorbance of the supernatant was measured at 485 nm using a spectrophotometer. Root dehydrogenase activity (µg·g_fw_^−1^·h^−1^) was calculated based on TTF content as follows:

Root dehydrogenase activity  =  (TTF content in extract  ×  total extract volume) ÷ (root massfw  ×  incubation time).

### 4.13 Animal Toxicity Assessment

#### 4.13.1 Earthworm toxicity assay

HOAc‐EB and EB were prepared as 10 000 mg·L^−1^ (A.I.) solutions in methanol. The solutions were mixed with 10 g of quartz sand to form test mixtures. The mixtures were placed in a fume hood until the methanol completely evaporated. Subsequently, the mixtures were incorporated into 500 g of soil to achieve A.I. concentrations of 1, 0.5, and 0.1 mg·kg^−1^. Deionized water was added to adjust the soil moisture content to 35% of dry soil weight. Forty earthworms were introduced into each treatment group. Earthworm mortality was recorded 5 days post‐exposure. The experiment was independently repeated three times. During the assay, earthworms were regularly inspected for activity, body integrity, and abnormal surface behavior, and soil moisture was maintained to minimize unnecessary stress. Individuals showing no spontaneous movement and no response to gentle tactile stimulation were recorded as dead and removed promptly. After the experiment, all surviving earthworms were humanely euthanized to minimize unnecessary suffering.

#### 4.13.2 Zebrafish Toxicity Assay

HOAc‐EB and EB were prepared as suspensions at five A.I. concentrations (0.0121, 0.0243, 0.04875, 0.0975, and 0.195 mg·L^−1^) in 500 mL volumes, with deionized water used as the control group. Thirty zebrafish were exposed to each suspension, and mortality was recorded at 24and 48 h post‐exposure. The experiment was independently repeated three times. During exposure, zebrafish were regularly monitored for swimming behavior, equilibrium, respiratory movement, and responsiveness to gentle stimulation. Fish showing severe and persistent distress, loss of equilibrium, prolonged immobility, or moribund condition were considered to have reached humane endpoints and were immediately removed and humanely euthanized. All procedures were conducted in accordance with the approved animal ethics protocol and the 3R principle.

#### 4.13.3 Mouse Toxicity Assay

HOAc‐EB and EB were prepared as 1 mL suspensions at A.I. doses of 100, 150, and 200 mg·kg^−1^ based on individual mouse body weight. The suspensions were administered via oral gavage using a gavage needle directly into the stomach of each mouse. The time to death was recorded for each mouse in all treatment groups. The experiment was independently repeated three times. Mice were closely monitored after gavage for posture, locomotor activity, respiration, feeding and drinking behavior, and other overt clinical signs. Animals showing severe and persistent distress, inability to ambulate, inability to access food or water, unresponsiveness, or a moribund state were considered to have reached humane endpoints and were immediately removed and humanely euthanized. Particular care was taken during gavage and observation to minimize unnecessary pain and distress.

### 4.14 Statistical Analyses

All statistical analyses were performed using Microsoft Excel and IBM SPSS Statistics 20.0 software. Differences between the two groups were analyzed using a two‐tailed unpaired Student's *t*‐test. Differences among multiple groups were analyzed using one‐way analysis of variance (ANOVA) followed by Tukey's post hoc test. Statistical significance was defined as *p* < 0.05. Different lowercase letters indicate significant differences at the 0.05 level. All data are presented as mean ± standard deviation (SD) from at least three independent experiments.

## Author Contributions

**Hanhong Xu**: conceptualization, methodology, software, writing review and editing, writing – original draft, resources, project administration, supervision, funding acquisition. **Lingda Zeng**: conceptualization, methodology, software, supervision, formal analysis, validation, investigation, visualization, writing original draft, writing – review and editing, project administration, data curation. **Jiaqi Wei**: conceptualization, investigation, writing – review and editing, writing – original draft, data curation, software, methodology, validation, formal analysis. **Jianxin Feng**: data curation, investigation, writing original draft, validation, formal analysis. **Huiting Zhu**: data curation, investigation, validation, formal analysis. **Kunzhong Lin**: data curation, investigation, validation, formal analysis. **Chenhan Liu**: data curation, investigation, validation, formal analysis. **Lin Li**: data curation, investigation, validation. **Zhuoyan Xiao**: data curation, investigation, validation, formal analysis. **Daihao Huang**: data curation, investigation, validation, formal analysis. **Rong Liu**: data curation, investigation, validation, formal analysis. **Huipeng Pan**: data curation, investigation, validation, formal analysis.

## Funding

This work was supported by the National Key Research and Development Program of China, 2024YFD1400100, and the Ministry of Agriculture and Rural Affairs of the People's Republic of China (Agricultural Science and Technology Major Project)

## Conflicts of Interest

Patent application related to this work has been granted (Patentee: South China Agricultural University; Name of inventors: Hanhong Xu, Lin Li, Lingda Zeng, Jiaqi Wei, Rong Liu; Patent No. ZL 2023 1 1007086.6). The remaining authors declare no competing interests.

## Supporting information




**Supporting File 1**: advs75914‐sup‐0001‐SuppMat.docx.

**Supporting File 2**: advs75914‐sup‐0002‐MovieS1.mp4.

**Supporting File 3**: advs75914‐sup‐0003‐MovieS2.mp4.

**Supporting File 4**: advs75914‐sup‐0004‐MovieS3.mp4.

## Data Availability

The data that support the findings of this study are available from the corresponding author upon reasonable request.
