# Supplementary material for: A Disaggregation Strategy for Nanopesticide Fabrication: Investigating the Impact of Nanosizing on Pesticide Biointeractions
Source: Adv Sci (Weinh). 2026 Jun 2:e75914. Online ahead of print. doi: 10.1002/advs.75914 (PMC13336719; doi:10.1002/advs.75914)
Supplement: Supplementary file 1 — Supporting File 1: advs75914‐sup‐0001‐SuppMat.docx. [file ADVS-9999-e75914-s003.docx]

**Supplementary Information**

**A Disaggregation Strategy for Nanopesticide Fabrication: Investigating the Impact of Nanosizing on Pesticide Biointeractions**

Jiaqi Wei^a^, Jianxin Feng^a^, Huiting Zhu^a^, Kunzhong Lin^a^, Chenhan Liu^a^, Lin Li^a^, Zhuoyan Xiao^a^, Daihao Huang^a^, Rong Liu^a^, Linda Zeng^a^*, and Hanhong Xu^a^*

a State Key Laboratory of Green Pesticide/Key Laboratory of Natural Pesticide and Chemical Biology, Ministry of Education, College of Plant Protection, South China Agricultural University; Guangzhou, 510642, China.

*Corresponding author.

1. mail addresses:

hhxu@scau.edu.cn (HanHong Xu);

zldvictor@163.com (Linda Zeng)

**Table of Contents**

Supplementary Figure 1. Effect of HOAc, hydrochloric acid, and phosphoric acid on the dispersion of EB 3

Supplementary Figure 2. Effect of formic acid, propionic acid, and n-butyric acid on the dispersion of EB 4

Supplementary Figure 3 Effect of ionic strength on the dispersion of EB 5

Supplementary Figure 4. Physical stability of HOAc-EB under different storage conditions 6

Supplementary Figure 5. Establishment of HPLC quantitative analysis method for EB. 7

Supplementary Figure 6. Chemical stability of HOAc-EB under different storage conditions 8

Supplementary Figure 7. Stability of HOAc-EB under concentration variations 9

Supplementary Figure 8. Mixing compatibility of HOAc-EB with pesticides (formulations) commonly co-formulated with EB 10

Supplementary Figure 9. Bioactivity of HOAc against *Megalurothrips usitatus* and *Meloidogyne enterolobii* 11

Supplementary Figure 10. Soil desorption of HOAc-EB and EB 13

Supplementary Figure 11. Phytotoxicity assessment of HOAc-EB and EB on cowpea with foliar spraying 14

Supplementary Figure 12. Phytotoxicity assessment of HOAc-EB and EB on chili pepper with root drenching 15

Supplementary Figure 13. Effects of HOAc-EB and EB on biochemical parameters in cowpea and chili pepper 16

Supplementary Figure 14. Biosafety assessment of HOAc-EB nanopesticide on zebrafish 17

**
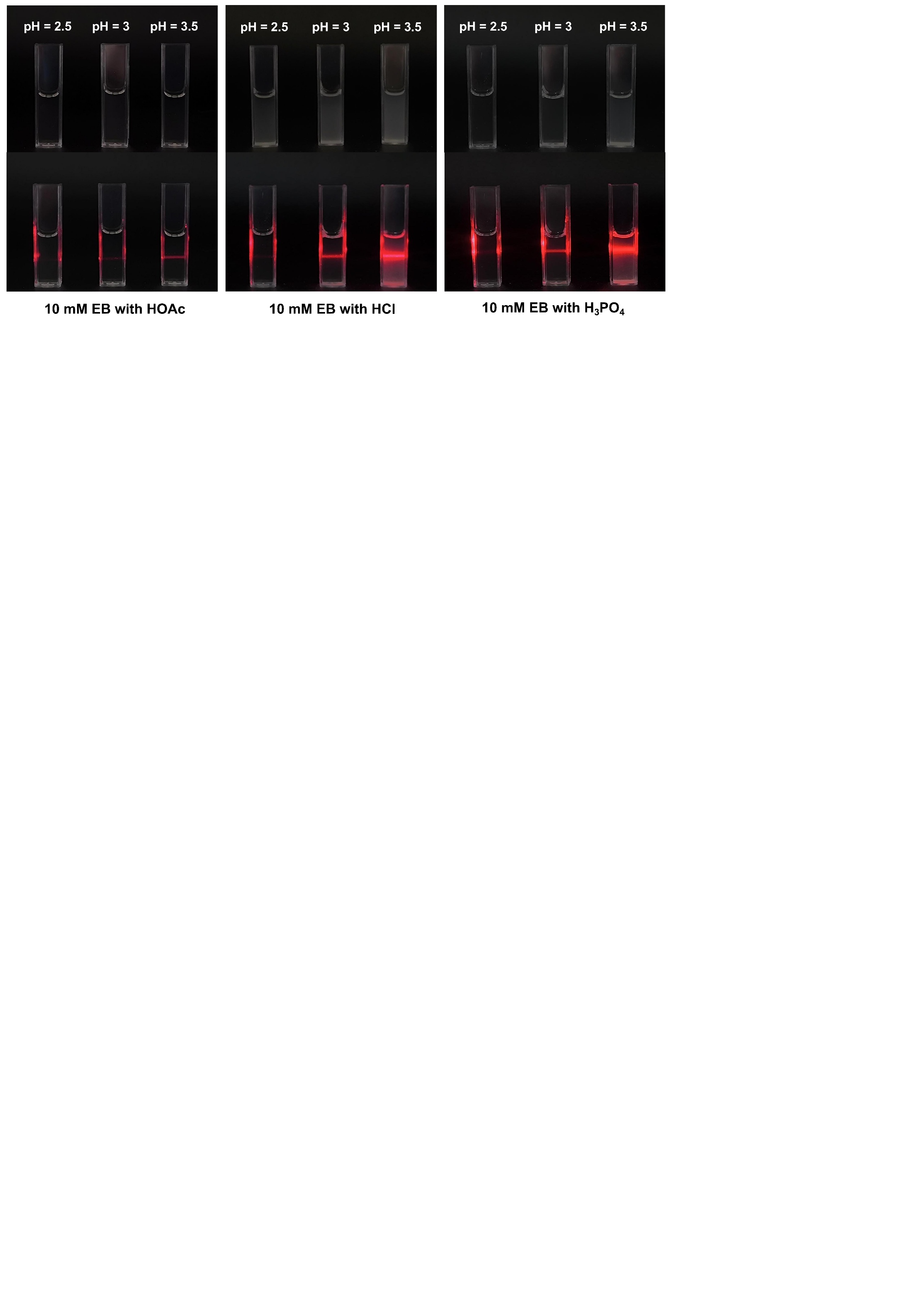
Supplementary Figure 1.** **Effect of HOAc, hydrochloric acid, and phosphoric acid on the dispersion of EB.** Photographs of EB (10 mM) with HOAc, hydrochloric acid and phosphoric acid at pH = 2.5, 3, 3.5.

**
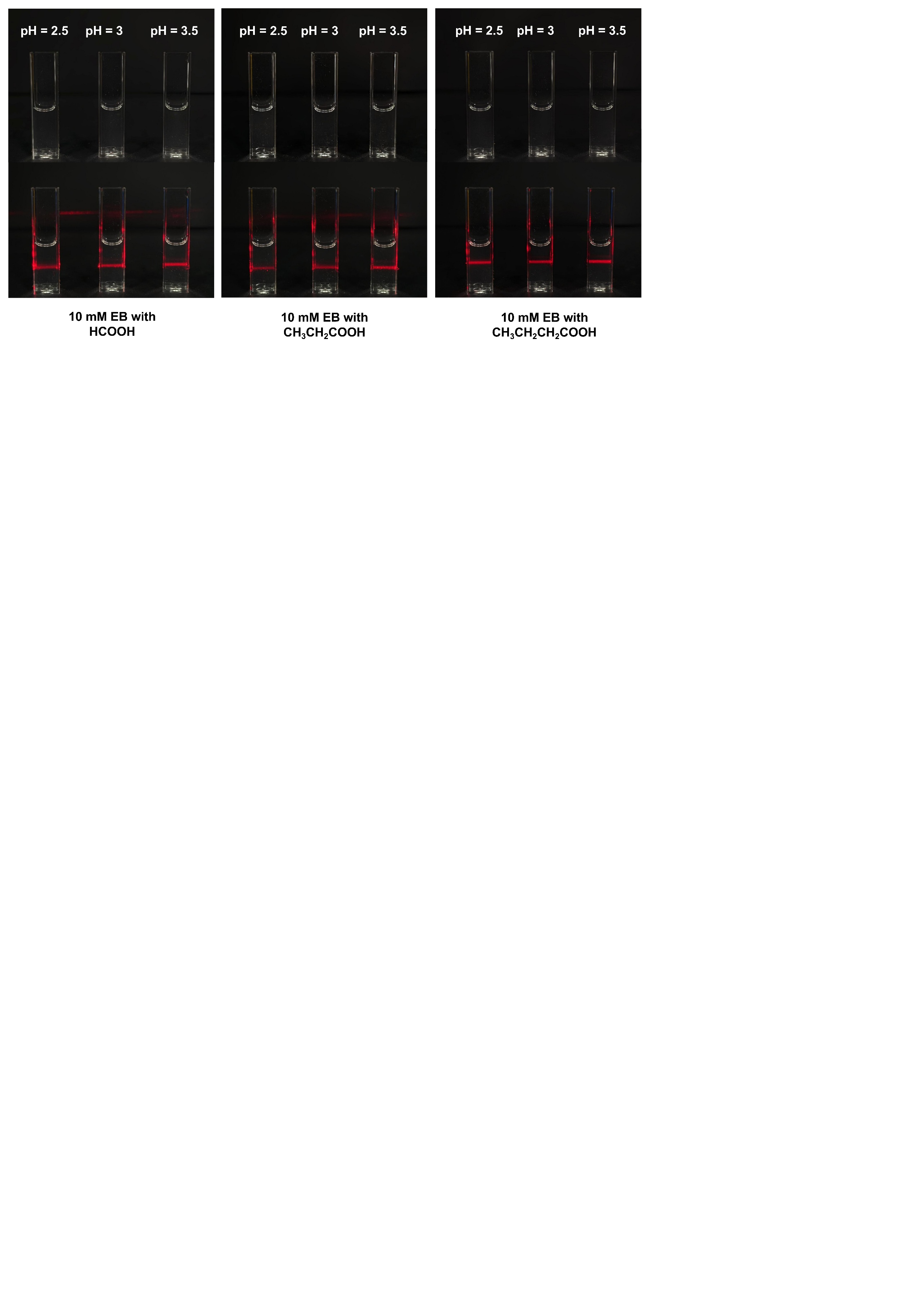
**

**Supplementary Figure 2.** **Effect of formic acid, propionic acid, and n-butyric acid on the dispersion of EB.** Photographs of EB (10 mM) with formic acid, propionic acid, and n-butyric acid at pH = 2.5, 3, 3.5.


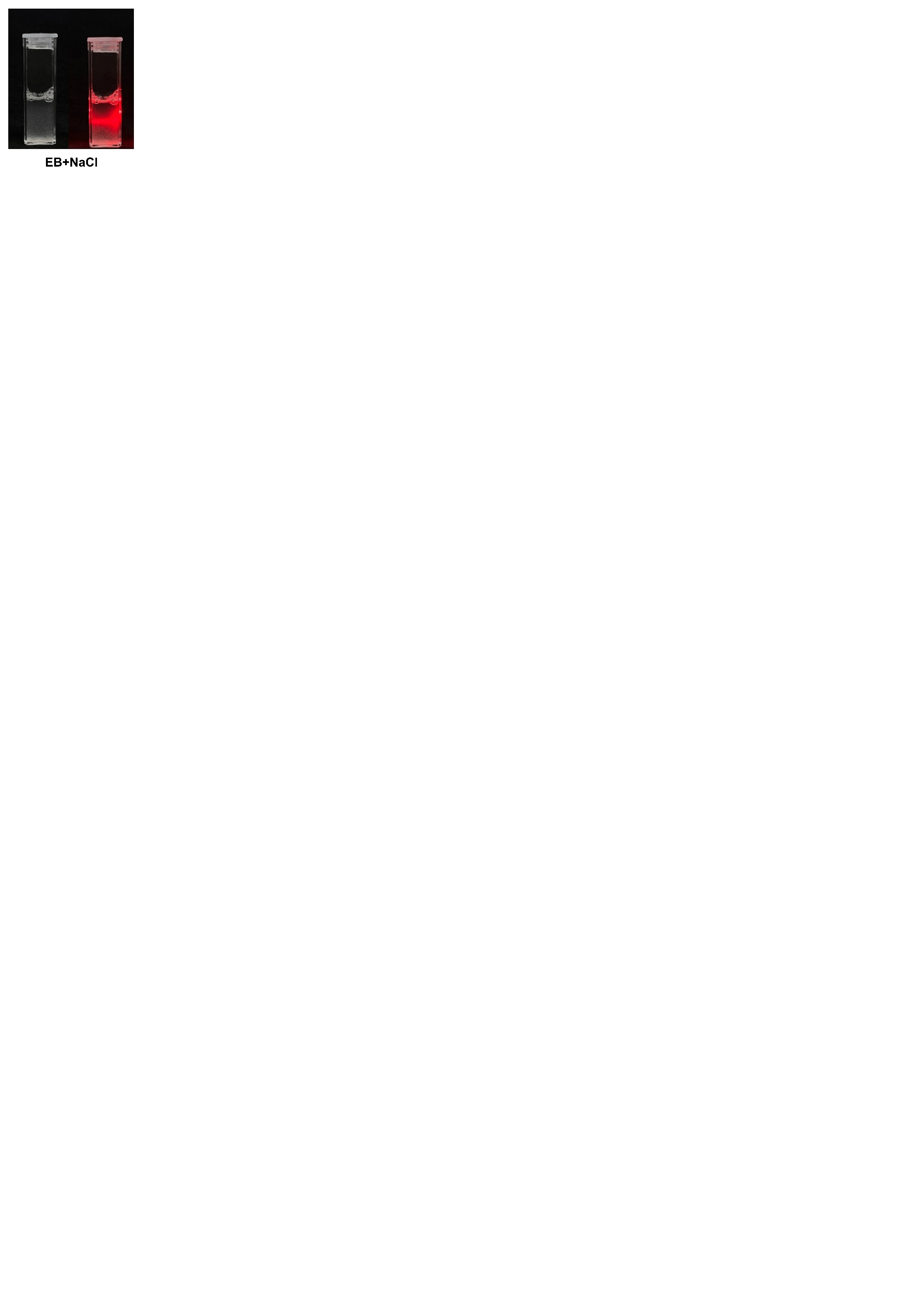


**Supplementary Figure 3. Effect of ionic strength on the dispersion of EB.** Photographs of EB (10 mM) with NaCl (20 mM).

**
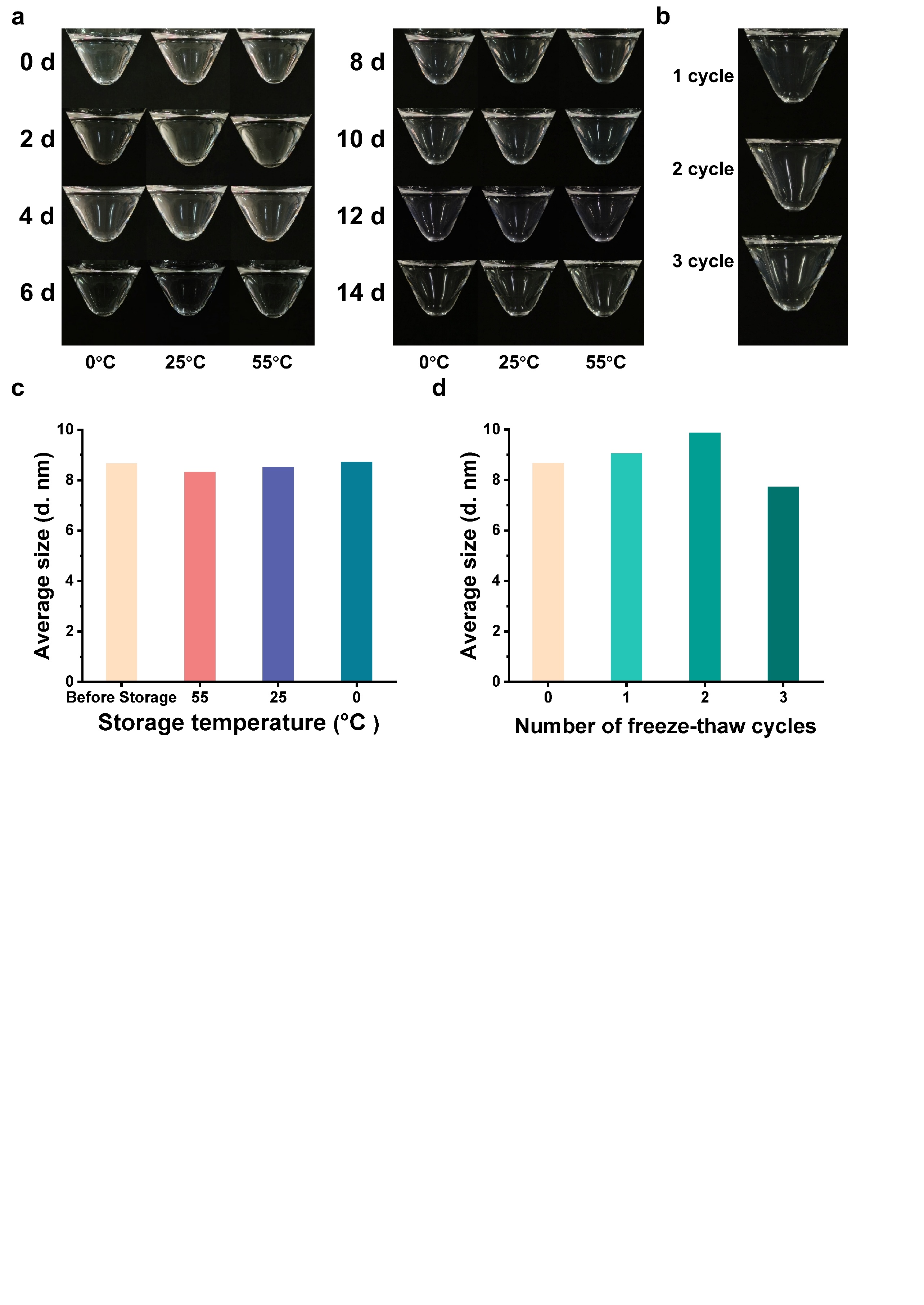
**

**Supplementary Figure 4. Physical stability of HOAc-EB under different storage conditions.** (a) Time-series photographs of HOAc-EB stored at different temperatures. (b) Photographs of HOAc-EB after multiple freeze-thaw cycles. (c) Variation in particle size of HOAc-EB stored at different temperatures for 14 days. (d) Variation in particle size of HOAc-EB after multiple freeze-thaw cycles.

**
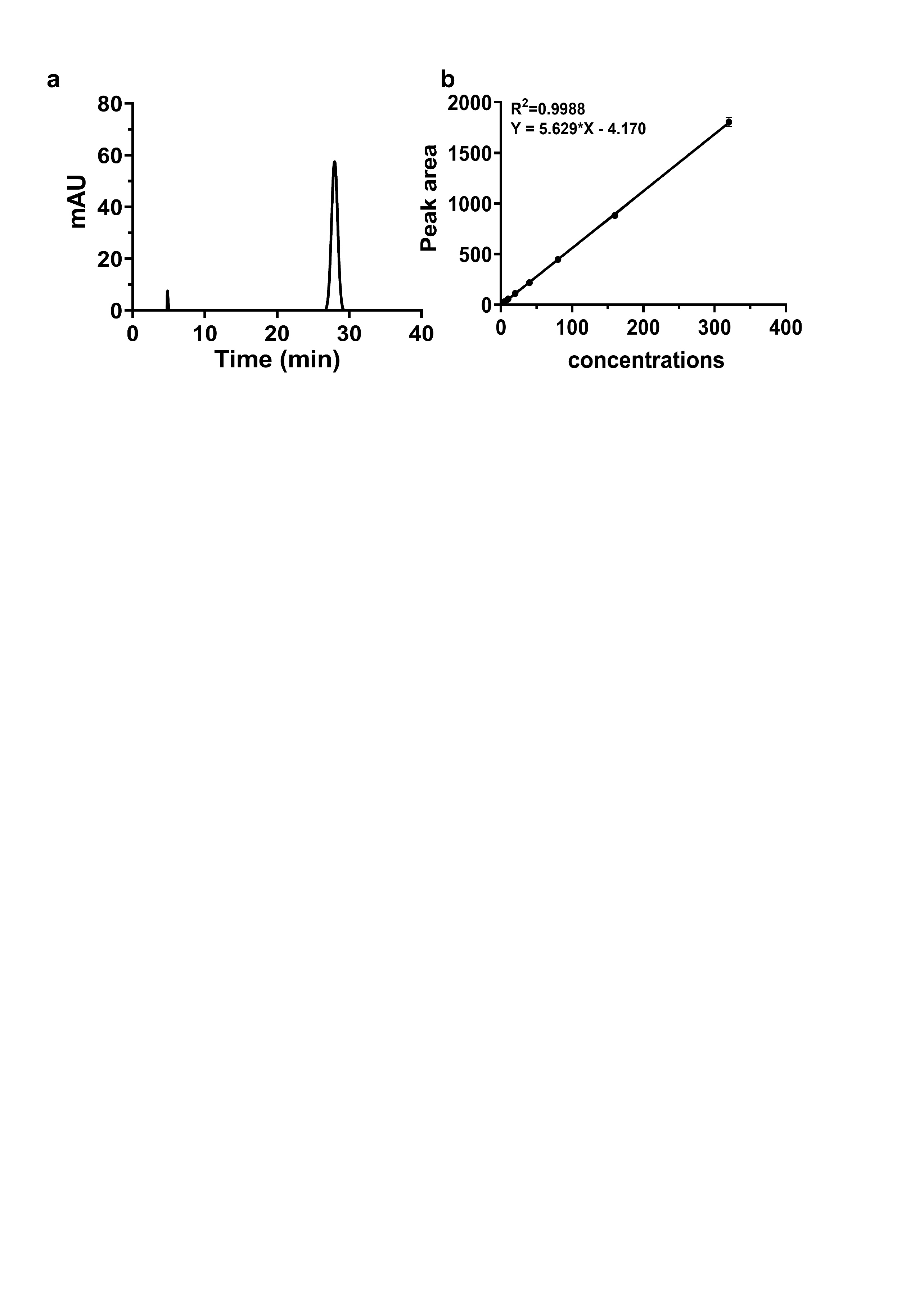
Supplementary Figure 5. Establishment of HPLC quantitative analysis method for EB.** (a) Typical chromatogram of EB. (b) HPLC calibration curve of EB.

To establish HPLC quantitative analysis method for EB, EB technical (95%, 0.1053 g) was accurately weighed and dissolved in HPLC-grade methanol to a final volume of 10 mL to obtain a 10,000 mg·L⁻¹ stock solution. A series of standard solutions (640, 320, 160, 80, 40, 20, and 10 mg·L⁻¹) were prepared by stepwise dilution with HPLC-grade methanol. All solutions were filtered through a 0.22 µm nylon membrane prior to analysis, and each concentration was analyzed in triplicate. A calibration curve was constructed by plotting peak area against concentration. The results showed that EB exhibited a single symmetric peak at approximately 27–28 min with good separation (Supplementary Figure 5a). A good linear relationship was observed between concentration and peak area over the range of 10–320 mg·L⁻¹ (y = 5.629x − 4.170, R² = 0.9988, Supplementary Figure 5b).


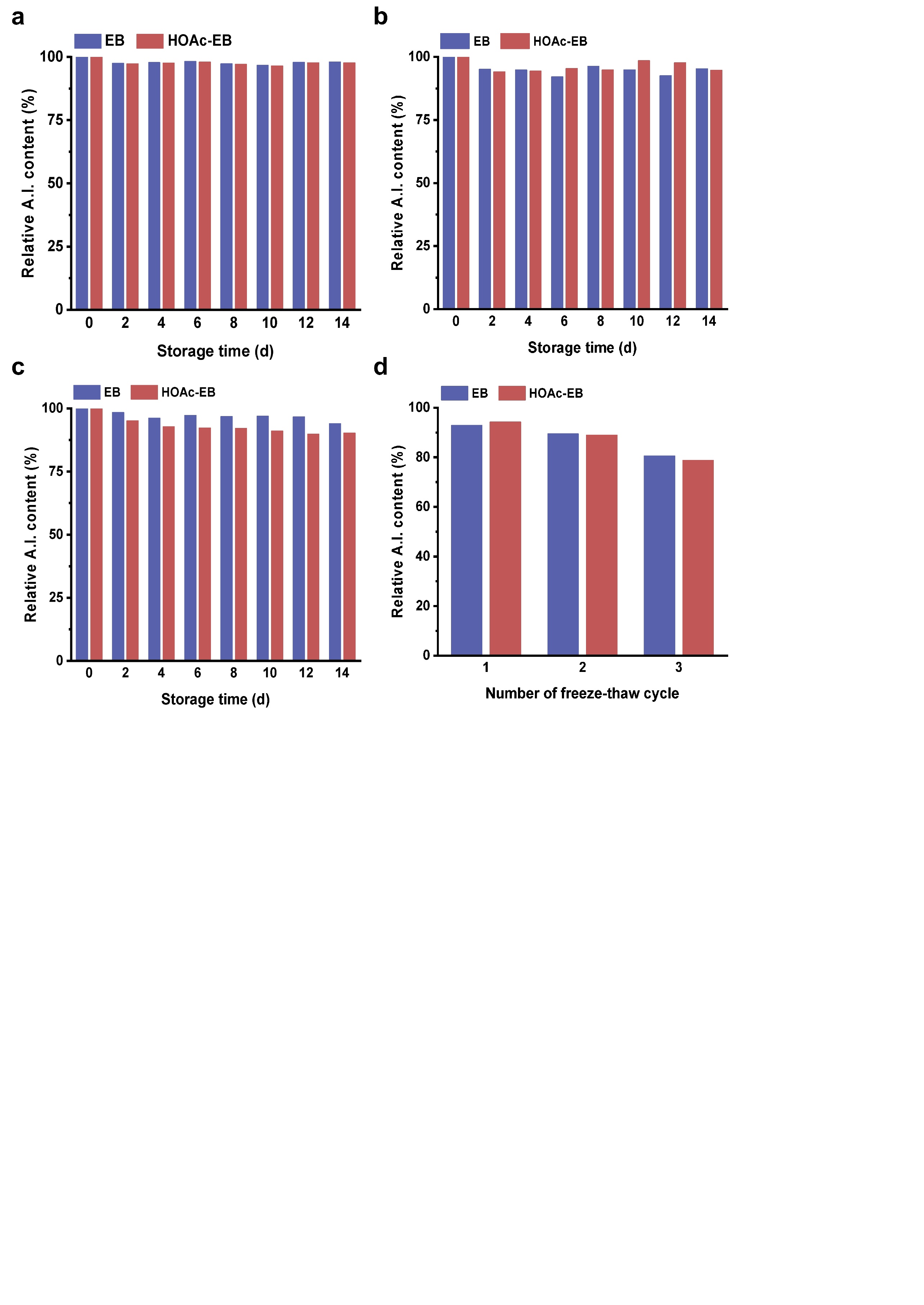


**Supplementary Figure 6. Chemical stability of HOAc-EB under different storage conditions.** Variation in relative A.I. content in EB and HOAc-EB stored at (a) 0°C, (b) 25°C, and (c) 55°C. (d) Variation in relative A.I. content in EB and HOAc-EB after multiple freeze-thaw cycles.

**
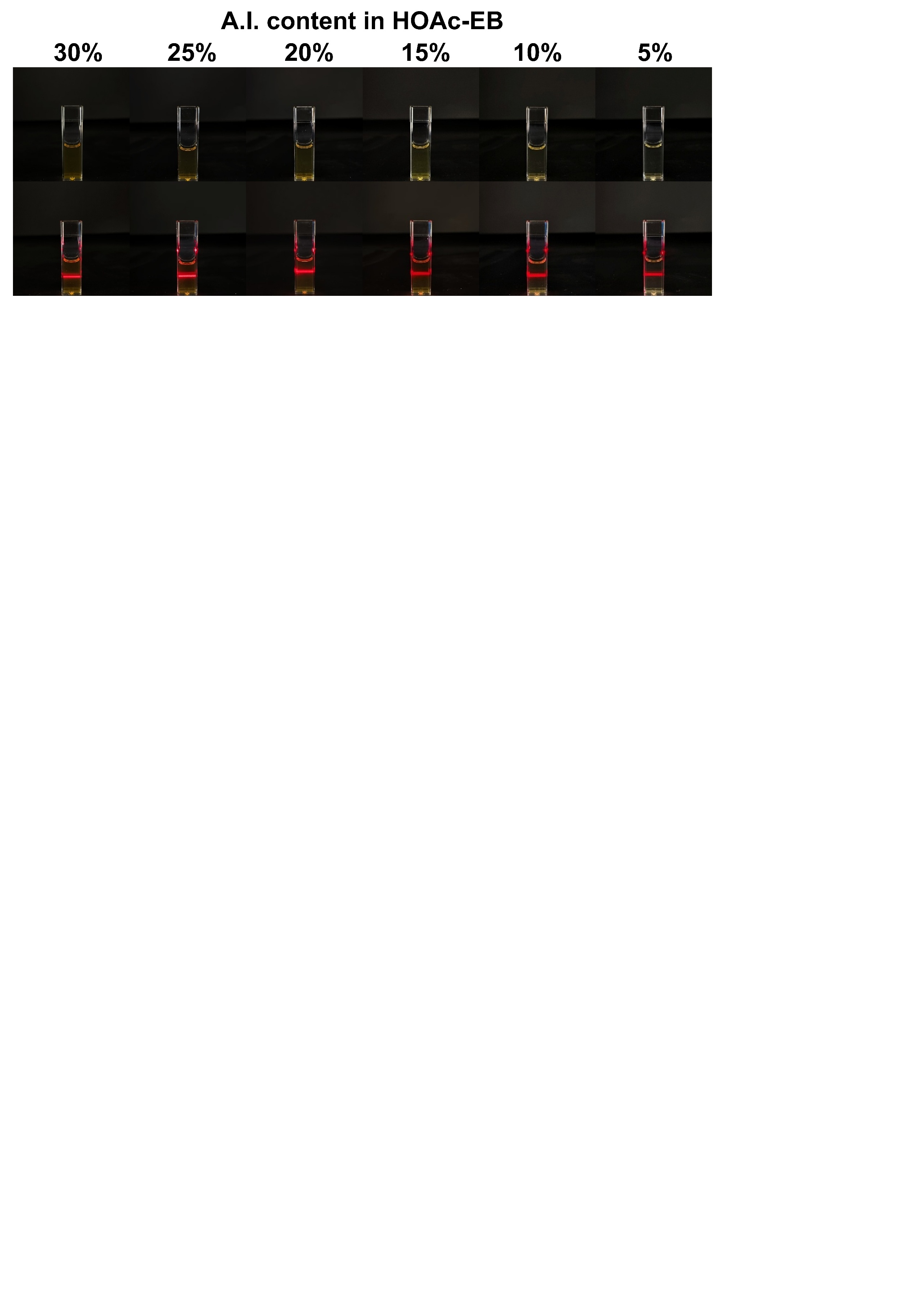
**

**Supplementary Figure 7. Stability of HOAc-EB under concentration variations.** Photographs of HOAc-EB with different A.I. content.

**
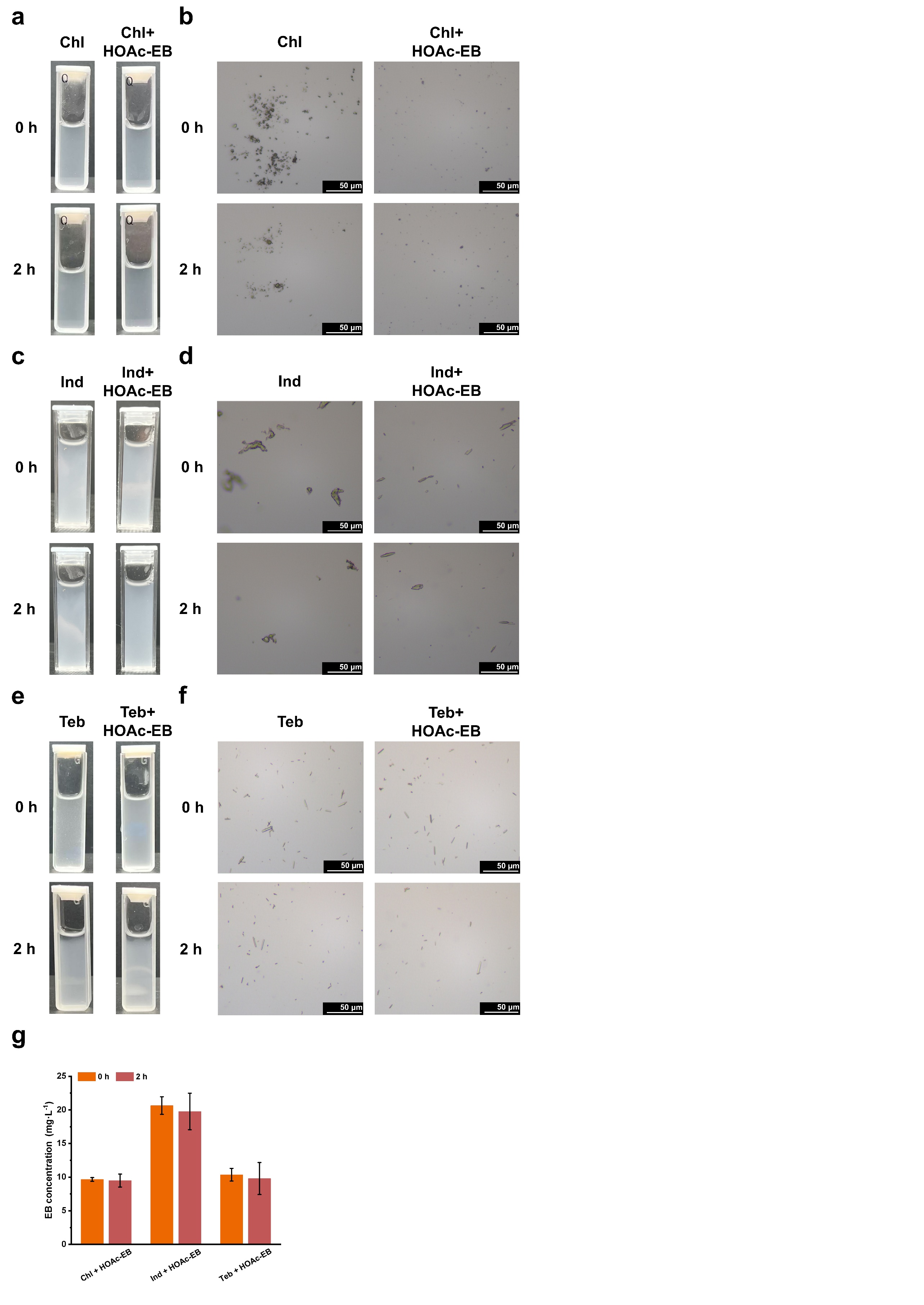
**

**Supplementary Figure 8.** **Mixing compatibility of HOAc-EB with pesticides (formulations) commonly co-formulated with EB.** (a) Photographs and (b) optical microscope images of chlorantraniliprole suspension (Chl, 0.003%) and Chl + HOAc-EB (0.003% + 0.001%) at 0 h and 2 h. (c) Photographs and (d) optical microscope images of indoxacarb suspension (Ind, 0.004%) and Ind + HOAc-EB (0.004% + 0.002%) at 0 h and 2 h. (e) Photographs and (f) optical microscope images of tebufenozide suspension (Teb, 0.004%) and Teb + HOAc-EB (0.004% + 0.001%) at 0 h and 2 h. (g) EB concentration in supernatants of Chl + HOAc-EB, ind + HOAc-EB and Teb + HOAc-EB at 0 h and 2 h.

In pesticide application, multiple pesticides are often co-applied through mixing. Therefore, mixing compatibility is of critical importance for nanopesticides. To evaluate the mixing compatibility of HOAc-EB with other pesticide formulations, suspension concentrate (SC) formulations of three pesticides commonly co-formulated with EB—chlorantraniliprole, indoxacarb, and tebufenozide—were selected for mixing with HOAc-EB. Three pesticide mixtures were prepared based on their typical co-formulation ratios with EB: (1) 9% chlorantraniliprole + 3% EB, (2) 2% indoxacarb + 1% EB, (3) 12% tebufenozide + 3% EB. Subsequently, the mixtures were diluted to field-recommended application concentrations.

The visual appearance of the diluted suspensions was observed at 0 h and 2 h, and the particle morphology was examined using an optical microscope. As shown in Supplementary Figure 8a–8f, no phase separation, visible sedimentation, or flocculation was observed in either the diluted pesticide SC alone or those mixing with HOAc-EB at both time points. Furthermore, microscopic images revealed that the addition of HOAc-EB did not alter the particle size or induce aggregation of the three pesticides. For EB, no precipitation of EB occurred after mixing with the three pesticide formulations and standing for 2 hours as shown by the variation on EB content in supernatants (Supplementary Figure S8g). These results indicate that HOAc-EB exhibits excellent mixing compatibility with other pesticide formulations.


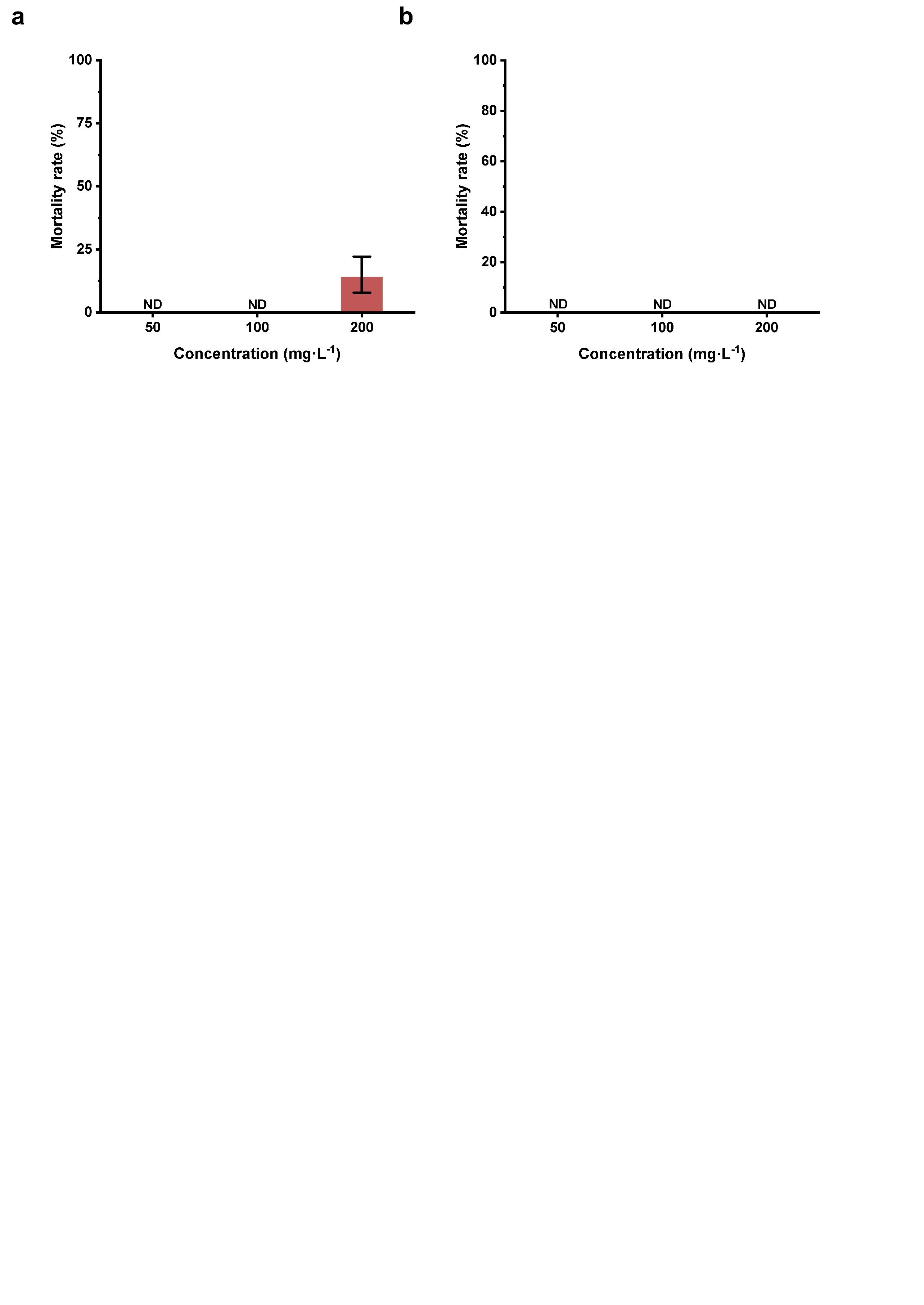


**Supplementary Figure 9.** **Bioactivity of HOAc against *Megalurothrips usitatus* and *Meloidogyne enterolobii*. a,b,** Dose-mortality relationship of HOAc against (a) *Megalurothrips usitatus* and (b) *Meloidogyne enterolobii*. Data represent mean ± SD. n = 3 independent experiments. ND, not detected.

**
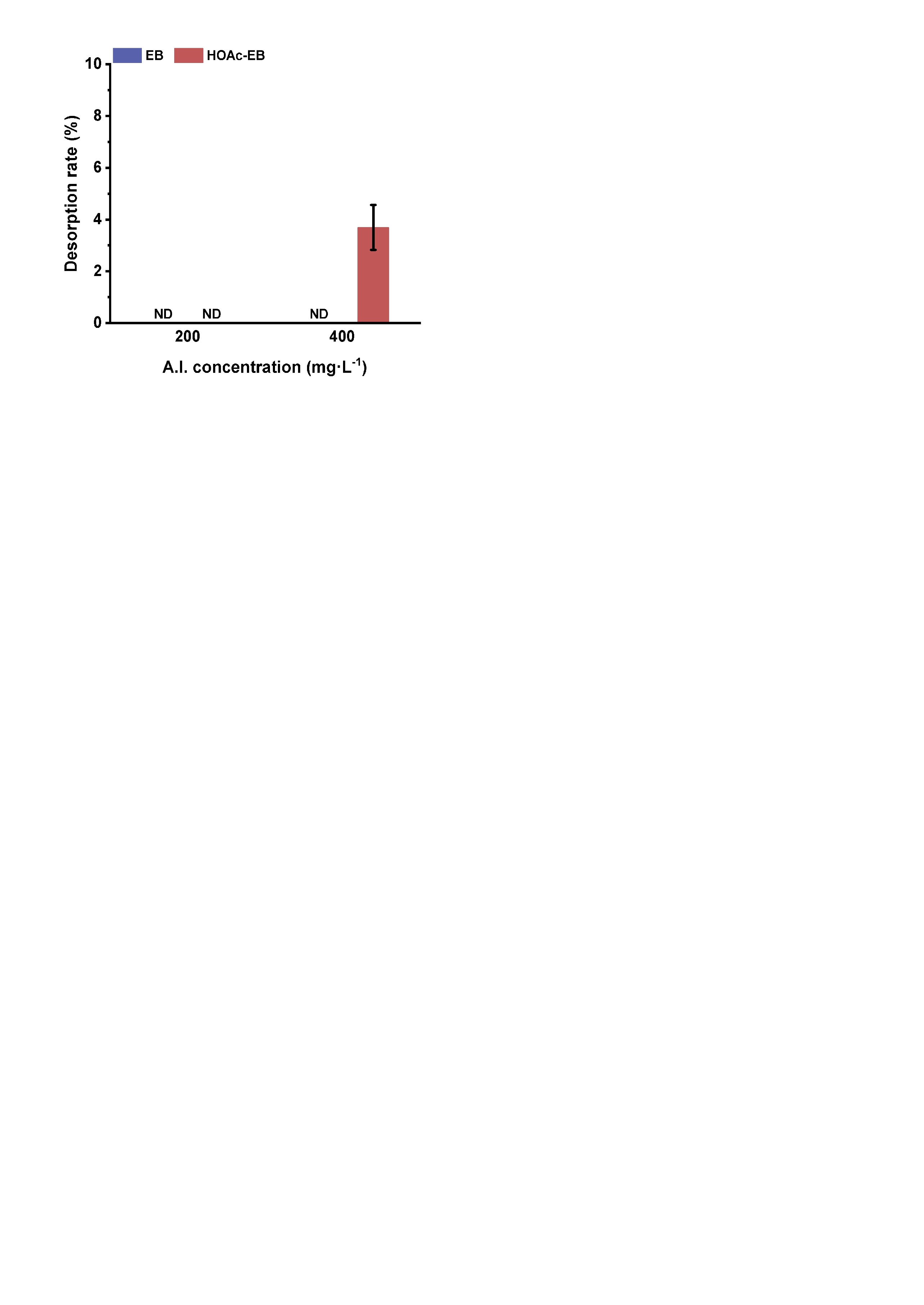
**

**Supplementary Figure 10. Soil desorption of HOAc-EB and EB.** Desorption rate of HOAc-EB and EB in soil with different A.I. concentration. Data represent mean ± SD. n = 3 independent experiments. ND, not detected.

**
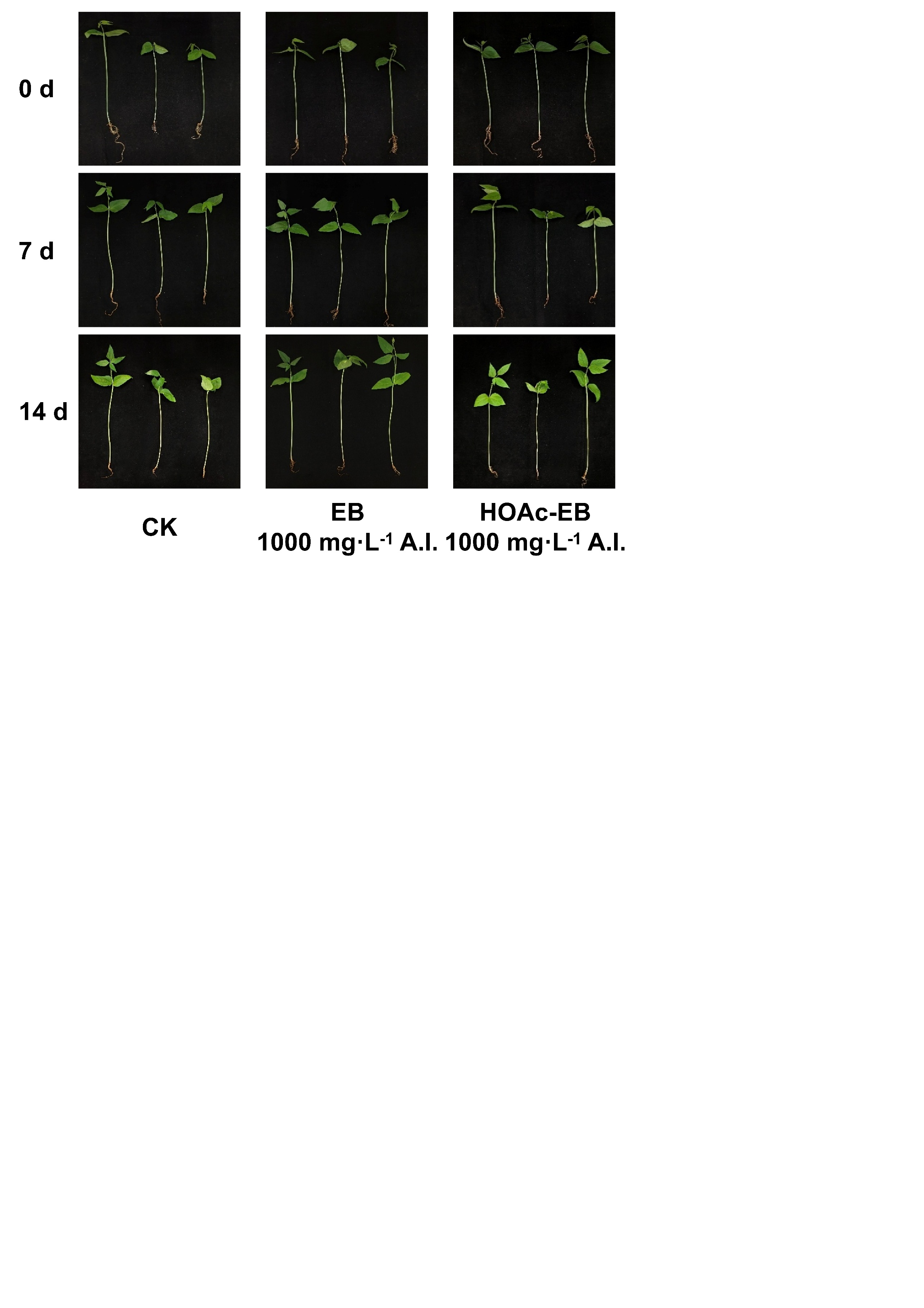
**

**Supplementary Figure 11. Phytotoxicity assessment of HOAc-EB and EB on** **cowpea with foliar spraying.** Photographs of cowpea plants sprayed with HOAc-EB, EB or water. Treatments were applied consecutively at 9:00 AM daily.

**
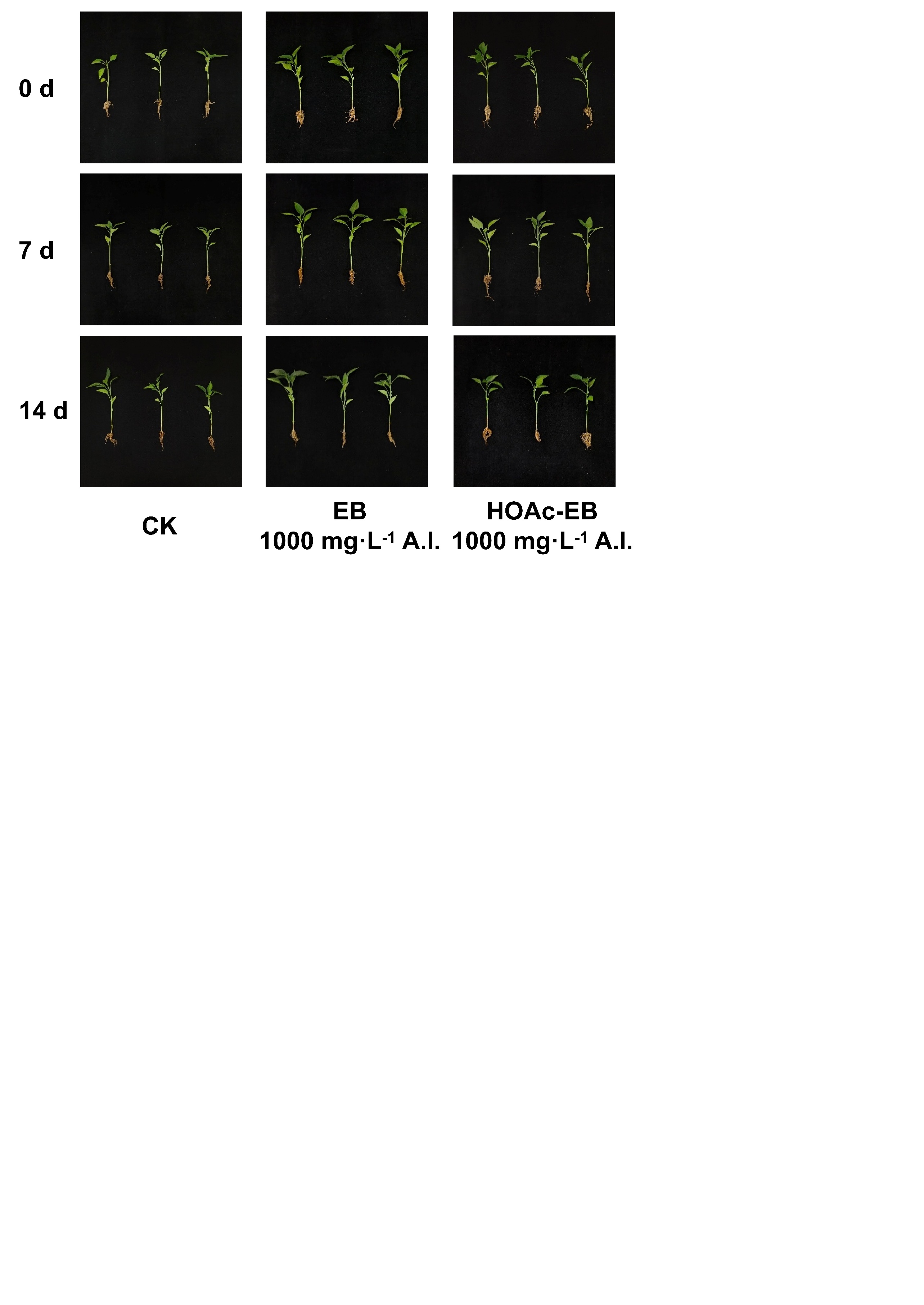
**

**Supplementary Figure 12. Phytotoxicity assessment of HOAc-EB and EB on chili pepper with root drenching.** Photographs of chili pepper plants root-drenched with HOAc-EB, EB or water. Treatments were applied at first day.

**
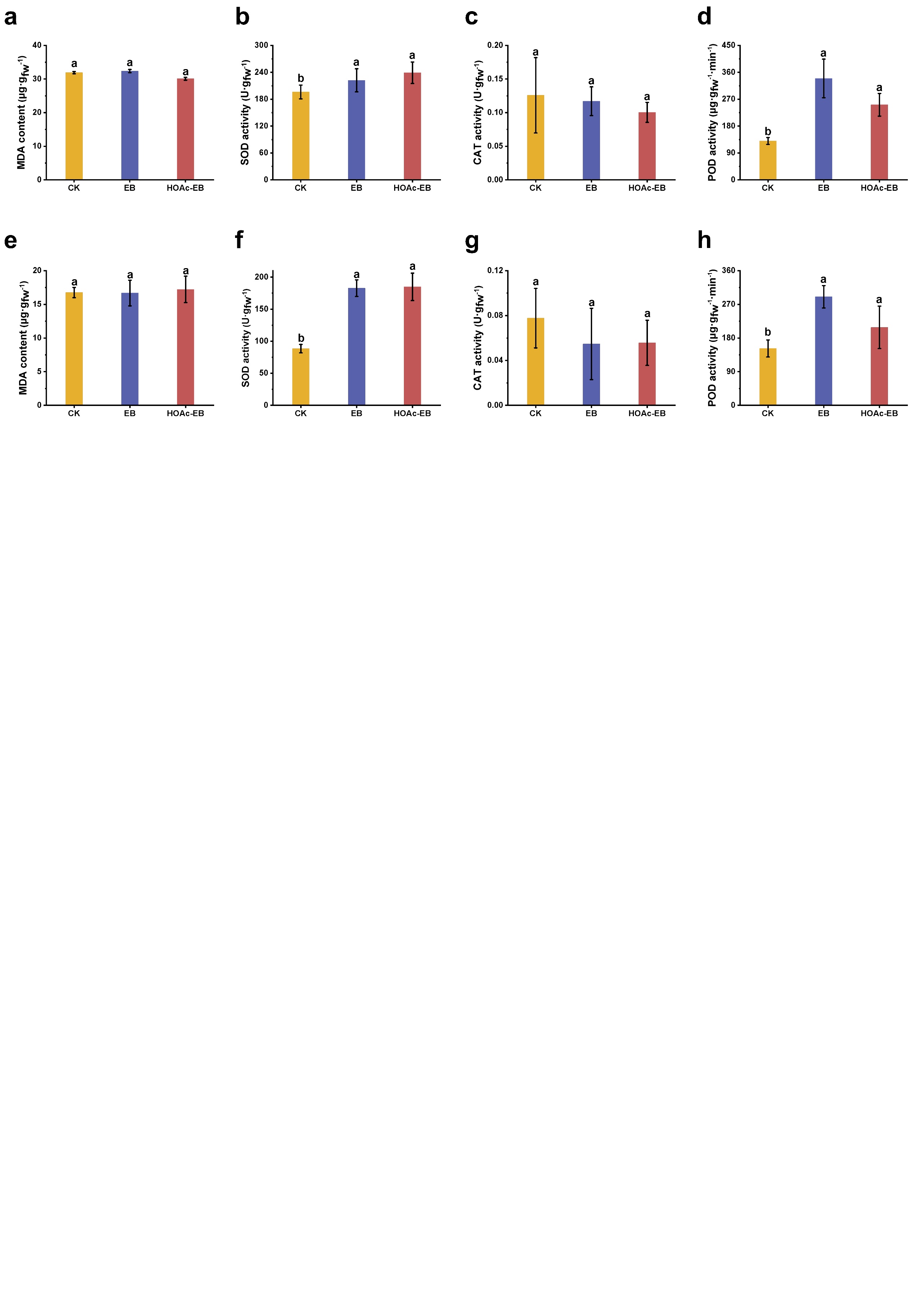
**

**Supplementary Figure 13. Effects of HOAc-EB and EB on biochemical parameters in cowpea and chili pepper.** (a) Malondialdehyde (MDA) content, (b) superoxide dismutase (SOD), (c) catalase (CAT), and (d) peroxidase (POD) activities in leaves of cowpea plants sprayed with HOAc-EB, EB or water for 14 days. Treatments were applied consecutively at 9:00 AM daily. (e) MDA content, (f) SOD, (g) CAT, and (h) POD activities in roots of chili pepper plants root-drenched with HOAc-EB, EB or water after 14 days. Treatments were applied on the first day. Differences were analysed using one-way ANOVA and Tukey's post hoc test, with different lowercase letters indicating significant differences (p < 0.05). Data represent mean ± SD. n = 3 independent experiments.

**
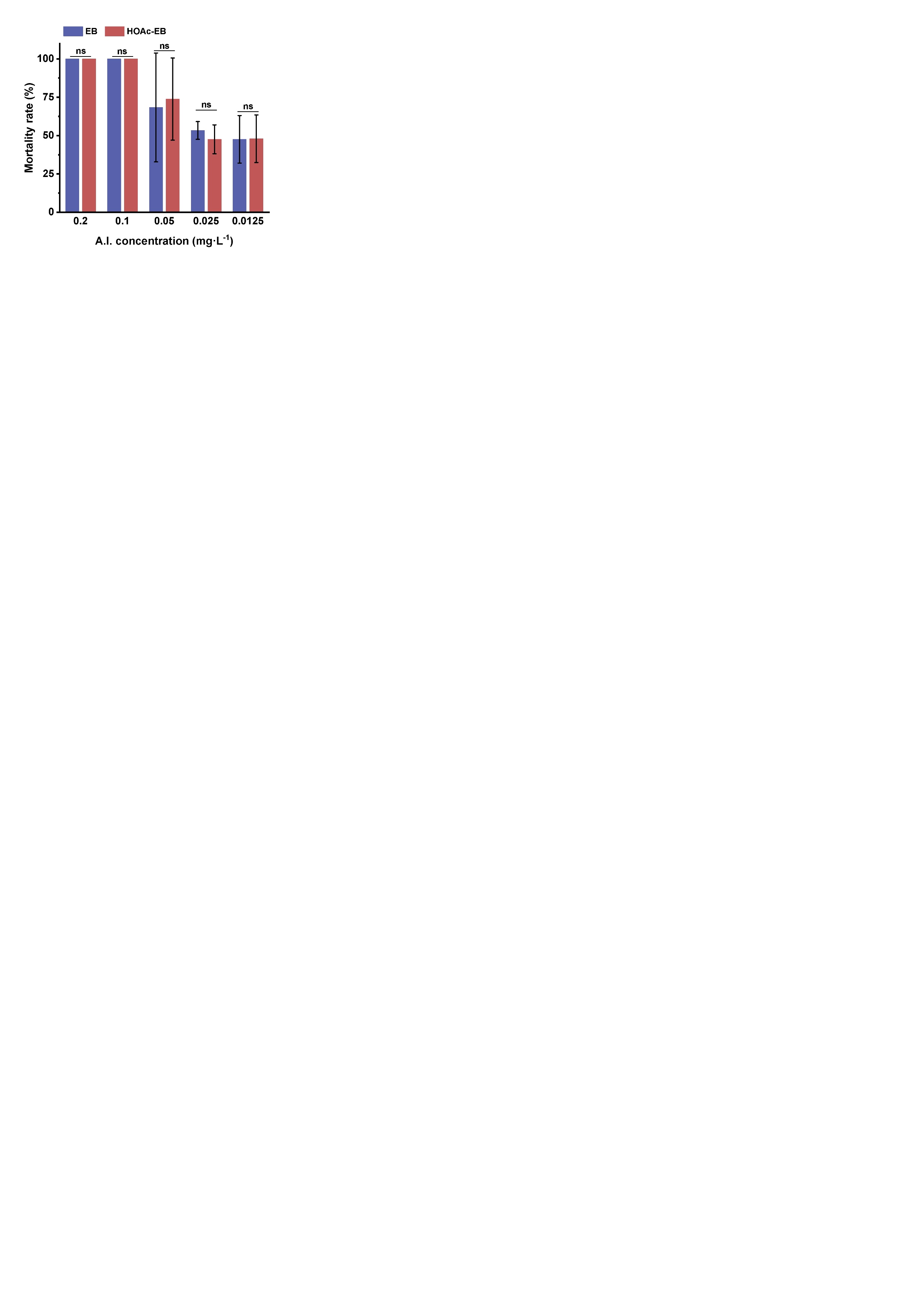
Supplementary Figure 14. Biosafety assessment of HOAc-EB nanopesticide on zebrafish.** Dose-mortality relationship of HOAc-EB and EB against zebrafish at 48 h. Differences were analysed using the two-tailed unpaired t-test. Data represent mean ± SD. n = 3 independent experiments. ns: *P* > 0.05, *: *P* < 0.05, **: *P* < 0.01, ***: *P* < 0.001
